# β-catenin-independent regulation of Wnt target genes by RoR2 and ATF2/ATF4 in colon cancer cells

**DOI:** 10.1038/s41598-018-20641-5

**Published:** 2018-02-16

**Authors:** Oksana Voloshanenko, Uwe Schwartz, Dominique Kranz, Benedikt Rauscher, Michael Linnebacher, Iris Augustin, Michael Boutros

**Affiliations:** 1 German Cancer Research Center (DKFZ), Division of Signaling and Functional Genomics and Heidelberg University, Department of Cell and Molecular Biology, Medical Faculty Mannheim, 69120 Heidelberg, Germany; 2 General Surgery, Section of Molecular Oncology and Immunotherapy, University of Rostock, 18057 Rostock, Germany

## Abstract

Wnt signaling is an evolutionarily conserved signaling route required for development and homeostasis. While canonical, β-catenin-dependent Wnt signaling is well studied and has been linked to many forms of cancer, much less is known about the role of non-canonical, β-catenin-independent Wnt signaling. Here, we aimed at identifying a β-catenin-independent Wnt target gene signature in order to understand the functional significance of non-canonical signaling in colon cancer cells. Gene expression profiling was performed after silencing of key components of Wnt signaling pathway and an iterative signature algorithm was applied to predict pathway-dependent gene signatures. Independent experiments confirmed several target genes, including *PLOD2*, *HADH*, *LCOR* and *REEP1* as non-canonical target genes in various colon cancer cells. Moreover, non-canonical Wnt target genes are regulated *via* RoR2, Dvl2, ATF2 and ATF4. Furthermore, we show that the ligands Wnt5a/b are upstream regulators of the non-canonical signature and moreover regulate proliferation of cancer cells in a β-catenin-independent manner. Our experiments indicate that colon cancer cells are dependent on both β-catenin-dependent and –independent Wnt signaling routes for growth and proliferation.

## Introduction

Wnt signaling pathways control a wide range of processes during embryonic development and tissue homeostasis. Deregulation of these pathways has been linked to many human diseases, including cancer^1,2^. Canonical Wnt signaling has been associated with breast^3–5^, colon^6^ and gastric cancer development^7,8^. Non-canonical, β-catenin-independent Wnt signaling is proposed to have a multifunctional role in tumorigenesis, being either tumor supportive or suppressive depending on tumorstage and tumortype^9^.

In mammals, the family of Wnt ligands consists of 19 members, which trigger multiple intracellular signaling cascades and orchestrate complex, context-dependent responses. Expression of Wnt ligands is tissue- and cell type specific^10,11^. Cells secrete Wnts in autocrine or paracrine manner with the help of two Wnt-secretion specific proteins: Porcupine (Porcn), an O-acyltransferase located in the endoplasmic reticulum (ER) responsible for Wnt palmitoylation^12,13^, and Evi/Wls/GRP177 which is as a cargo-receptor required for transport of palmitoylated Wnt proteins ER to Golgi and then to the extracellular space^14–17^.

Wnt ligands can induce multiple pathways depending on the available receptors^18^ and the presence or absence of enhancers or inhibitors of signaling, such as members of the R-spondin, SFRP and Dickkopf (DKK) families^19,20^. While Wnt1, Wnt3 and Wnt3a mostly activate a β-catenin-dependent signaling pathway^21^, Wnt5a/b and Wnt11 primarily induce non-canonical, β-catenin-independent pathways^22,23^. However, predicting the signaling outcome of different Wnt ligands has remained difficult.

Binding of canonical Wnt proteins to Frizzled (FZD) receptors and the LRP5/6 co-receptors leads to the relocation of Dishevelled (Dvl), a multi-domain scaffolding protein to the plasma membrane^20,24^. Recruitment of additional factors, including kinases, leads to phosphorylation of LRP6. In the absence of Wnt ligands β-catenin is constantly degraded by a destruction complex containing adenomatous polyposis coli (APC)^25^, a tumorsuppressor and negative regulator of Wnt signaling, the scaffold protein Axin1 and the kinases GSK3β and CK1α^26,27^. These kinases phosphorylate β-catenin, leading to its ubiquitylation and proteasomal degradation. Axin1 and GSK3β are recruited to the receptor complex upon Wnt stimulation and this leads to a breakdown of the destruction complex and β-catenin stabilization. β-catenin then translocates to the nucleus where it forms a complex with TCF/LEF transcription factors and activates Wnt target gene expression^28,29^.

Non-canonical, β-catenin independent pathways, such as planar cell polarity (PCP)^30,31^, Wnt/Ca^2+^^2,32^, RoR2/JNK signaling^33^, are less understood^34^. In mouse, RoR1/2 and Wnt5a knockouts show comparable phenotypes during development^10^ and blastocyst attachment and implantation^35^. Dvl, an adaptor protein composed of a DIX, PDZ and DEP domain, is required for both non-canonical and canonical Wnt signaling^36^. Its DIX domain is required for canonical Wnt signaling, the DEP domain links receptor activation to non-canonical pathways^37–39^. FZDs, RoR1/2^40,41^, Ryk^42^ and PTK7^43^ transmembrane proteins have all been proposed as non-canonical Wnt receptors depending on the model or cellular system.

Downstream effectors of the Wnt/PCP pathway are Rac, RhoA and JNK, linking signaling at the membrane to AP1 and ATF2 transcription factors^37,44–47^. Rac1 and RhoA were reported to activate JNK after pathway stimulation by Wnt5a^48^. Wnt5a and Wnt11 binding to FZD and/or RoR2 also can lead to intracellular Ca^2+^ release, which activates Ca^2+^/calmodulin-dependent kinase II (CaMKII) and protein kinase C (PKC)^32^.

To date, the functional role and potential transcriptional targets of non-canonical, β-catenin-independent Wnt signaling in colon cancer cells is not well understood. In order to identify targets, we first performed RNAseq analysis of colon cancer cells upon silencing of several key Wnt signaling components. We then applied an iterative signature algorithm to identify genes, which are regulated by a Wnt cargo receptor Evi/Wls, independent of β-catenin and APC. Several identified genes were confirmed by independent methods and shown to be targets of a non-canonical RoR2/Dvl2/ATF signaling. Additionally, we found that these genes are regulated by auto- and paracrine Wnt5a/b secretion in colon cancer cells. We further demonstrate that Wnt5a/b is required for proliferation and viability of colon cancer cells *via* a β-catenin-independent signaling pathway. Taken together, these results indicate that survival of colon cancer cells is dependent on both canonical and non-canonical Wnt signaling.

## Results

### Prediction of non-canonical Wnt target genes by perturbation-based gene set analysis

**Figure 1:**
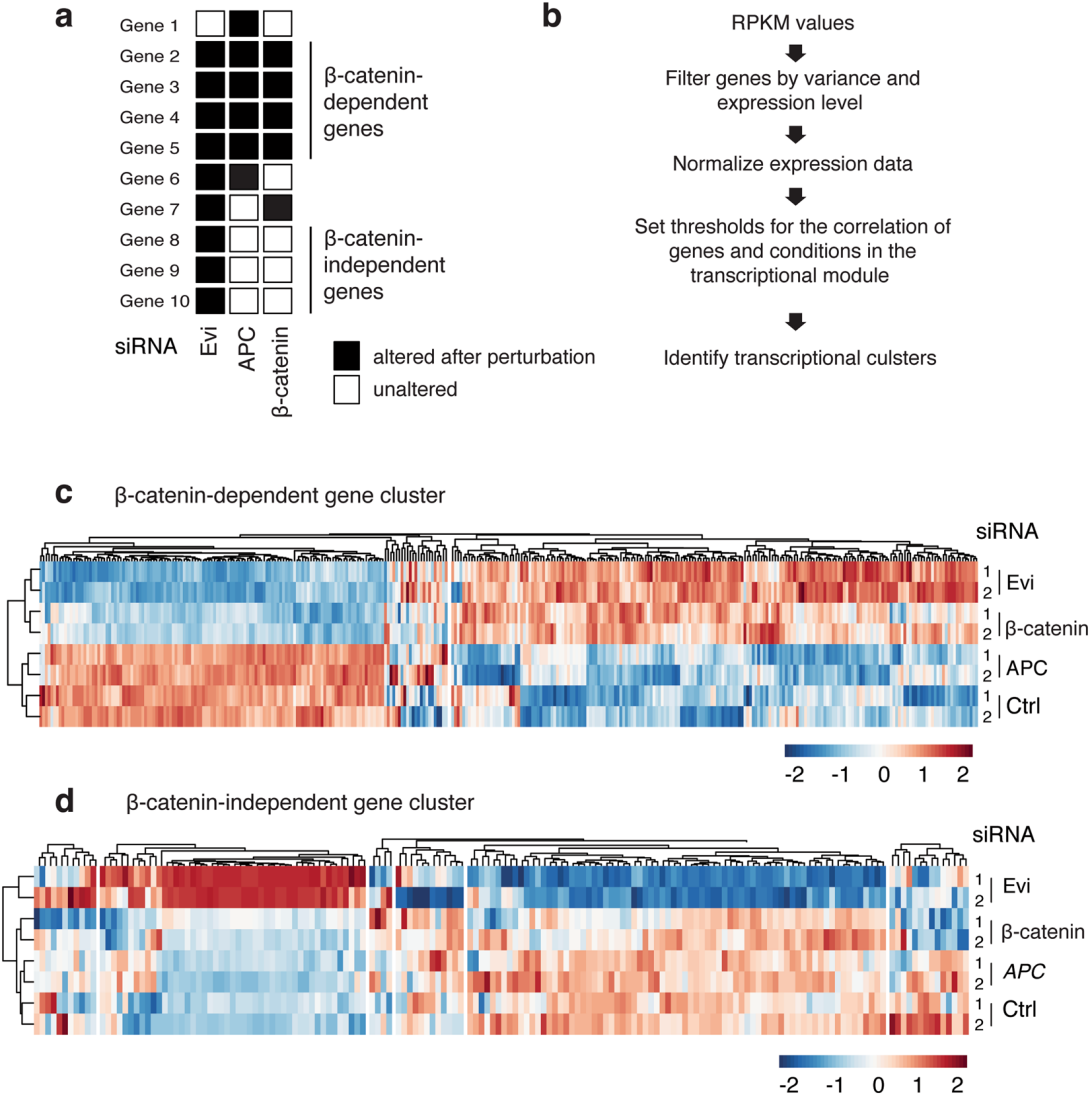
Identification of Evi/Wls-regulated Wnt non-canonical target genes. (**a**) Schematic representation of Evi/Wls-regulated genes and their separation regarding β-catenin dependency and independency. Rows represent diversity of target genes (E_n_) and columns indicate different knockdown conditions using siControl/Ctrl and siRNAs targeting *EVI/WLS*, *APC* or *CTNNB1/*β-catenin. (**b**) The steps of bioinformatical analyses for identification of meaningful biological clusters on the basis of RNAi RNAseq HCT116 data. (**c,d**) Representative clusters generated by ISA (Iterative Signature Algorithm), which include all 4 siRNAs. Upper (c) cluster shows β-catenin dependent Evi/Wls-regulated genes. (d) This cluster consists of β-catenin independent Evi/Wls-regulated target genes.

In order to characterize the role of β-catenin-independent signaling in colon cancer, we devised perturbation experiments to identify gene sets that are dependent on auto-paracrine Wnt secretion, but unaffected by depletion of β-catenin or APC^49^. After siRNA silencing of APC, β-catenin/*CTNNB1* and Evi/Wls, changes in the transcriptome of HCT116 colon cancer cells were analyzed by RNA sequencing (RNAseq) (Supplementary Fig. 1a). Figure 1a schematically illustrates expected target gene groups, which are dependent on Evi/Wls secretion in order to distinguish β-catenin-dependent *versus* independent gene sets. To identify such gene sets, we applied an iterative signature algorithm (ISA) to the transcriptome data (see Methods, R-script).

ISA is a biclustering approach, which determines co-regulated gene sets and their driving conditions without any *a priori* knowledge (Fig. 1b, Supplementary Fig. 1b)^50^. Applied to our RNAseq data, the iterative algorithm revealed 23 related clusters. In contrast to randomized data, clusters generated from the experimental data consist of small gene sets, which were co-regulated across conditions presented in this study (Supplementary Fig. 1a,c). Furthermore, replicates of the same perturbation condition were sub-clustered together within each set confirming the robustness of the approach (Fig. 1c,d, Supplementary Fig. 2). Four clusters exhibited a coherent expression pattern across all siRNA conditions and were therefore considered as Wnt-responsive gene sets (Supplementary Fig. 1c). Three clusters represented gene sets, which were regulated upon downregulation of β-catenin (Fig. 1c; Supplementary Fig. 2). The expression pattern coincides with targets of canonical, β-catenin-dependent signaling, consistent with our previous data showing that Evi/Wls silencing led to downregulation of canonical Wnt target genes in HCT116 cells^49,51^.

Genes in the fourth cluster changed their expression upon perturbation of Evi/Wls but not of β-catenin. These genes were potential targets that rely on non-canonical, β-catenin-independent Wnt signaling (Fig. 1d, Supplementary Table 1). Taken together, with this approach we identified candidate genes, which were regulated by non-canonical Wnt signaling in direct or indirect way.

### Confirmation of β-catenin-independent Wnt target genes

**Figure 2:**
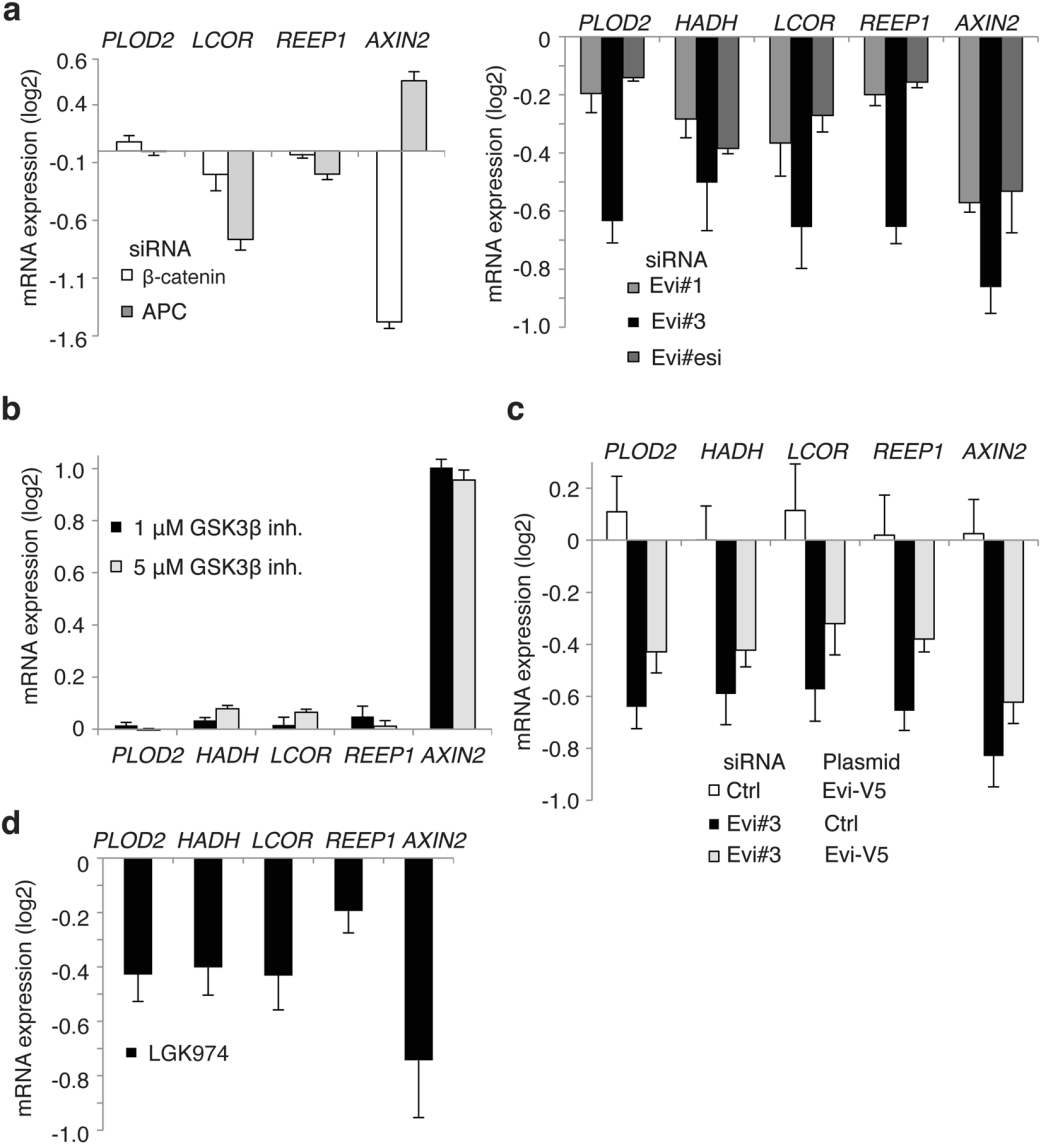
Non-canonical Wnt target genes are regulated by Evi/Wls but not by β-catenin or APC. (**a**) Silencing of Evi/Wls reduces both canonical and non-canonical target genes, whereas β-catenin and APC silencing effects the level of *AXIN2*, the classical canonical Wnt target gene. HCT116 cells were reverse transfected with the indicated siRNA and assessed for the expression of the indicated genes by qPCR 72 hours later. (**b**) Induction of canonical β-catenin signaling by GSKβ inhibitor XVI (CHIR99021) induces *AXIN2* but does not change mRNA levels of identified non-canonical target genes. HCT116 cells were treated with indicated amounts of the inhibitor for 24 hrs, then indicated genes were analyzed by qPCR. (**c**) Downregulation of non-canonical Wnt target genes is a specific effect of Evi/Wls silencing. Overexpression of Evi-V5 partly rescues the phenotype induced by siEvi#3. HCT116 cells were reverse transfected with indicated siRNAs and 24 hours later cells were transfected with pcDNA Ctrl *versus* Evi-V5. 48 hrs after the plasmid transfection the indicated genes were analyzed by qPCR. (**d**) HCT116 cells were treated with LGK974 for 96 hrs and identified non-canonical genes were analyzed by qPCR. *AXIN2* was used as positive control. (a-d) Every independent experiment was normalized to siCtrl (siRNA silencing experiments) or DMSO/Ctrl (drug treatment) and log_2_ transformed of relative quantitation (RQ). Results of 3–6 independent experiments are shown as mean ± s.e.m. *p* values of every condition compared to control using single samples *t*-test (**a,b,d**) or paired Student’s *t*-test (**c**) as well as values of independent experiments are shown in the Table S2.

Next, we focused our transcriptional analysis on genes, which expression was dependent on Evi/Wls, but not affected by depletion of APC or β-catenin/*CTNNB1*. In order to confirm Wnt signal dependent regulation, differential mRNA expression of *PLOD2*, *LCOR* and *REEP1* was analyzed by RT-qPCR after silencing by siRNAs against APC, β-catenin/*CTNNB1* and Evi/Wls (Fig. 2a and Supplementary Fig. 3a,b,d). As shown in Fig. 2a whereas silencing of APC and β-catenin/*CTNNB1* strongly affected *AXIN2* expression, the mRNA levels of *PLOD2*, *LCOR* and *REEP1* were not changed in the similar manner. However, *PLOD2*, *HADH*, *LCOR* and *REEP1* mRNA expression levels were reduced upon Evi/Wls silencing (Fig. 2a and Supplementary Fig. 3a,b). Additionally, we induced canonical Wnt signaling by treating HCT116 cells with GSK3β inhibitor (CHIR99021) for 24 hrs. Activation of canonical Wnt signaling revealed up-regulation of *AXIN2* mRNA expression similar to silencing of APC, when mRNA expression of non-canonical Evi/Wls target genes was not effected (Fig. 2b). In line with Fig. 2a silencing of Evi/Wls with an inducible short-hairpin RNA construct in HCT116 cells led to *PLOD2* downregulation (Supplementary Fig. 4a). Observed downregulation of *PLOD2*, *HADH, LCOR* and *REEP1* genes upon perturbation of Evi/Wls with siRNA was rescued by expression of a transgenic Evi-construct (Fig. 2c). These results indicate that the identified target genes relied on the autocrine release of Wnt ligands, but were independent of β-catenin-dependent signaling.

We asked whether we could phenocopy the genetic depletion of Evi/Wls by blocking palmitoylation^52,53^ and thereby secretion of Wnt proteins using the Porcupine inhibitor LGK974, which potently blocks secretion of all canonical and non-canonical Wnt ligands^54^. To this end, HCT116 cells were treated with LGK974 and expression of identified non-canonical Wnt target genes was assessed by qRT-PCR. As shown in Fig. 2d, mRNA expression of *PLOD2*, *HADH*, *LCOR*, *REEP1* and *AXIN2* genes were reduced, supporting a Wnt-dependent regulation.

**Figure 3:**
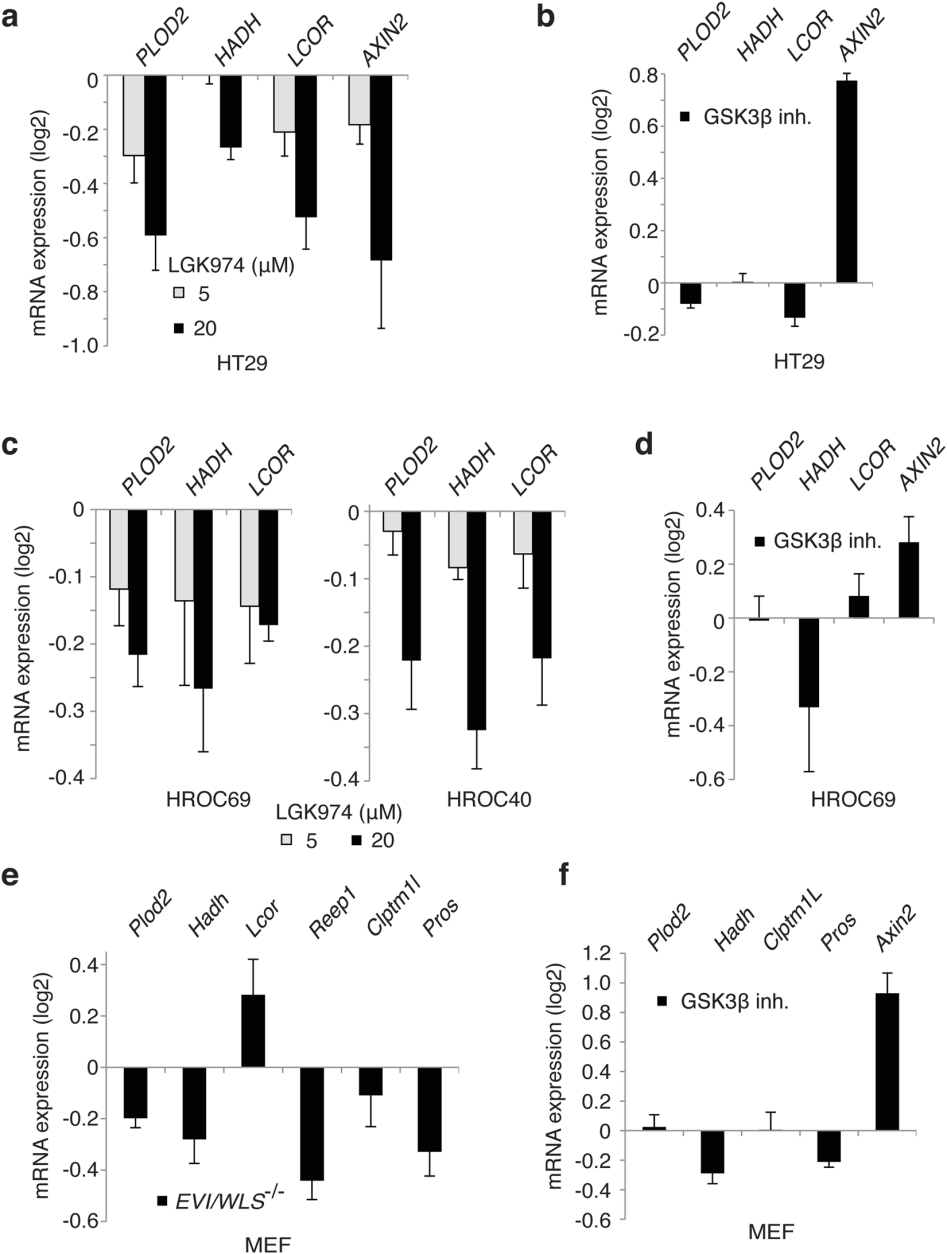
Non-canonical Wnt genes are regulated by Evi/Wls and porcupine in different cellular systems. (**a,b**) HT29 colon cancer cells were treated with the indicated amounts of LGK974 porcupine inhibitor for 96 hrs (a) or with 5 μM GSK3β inhibitor XVI (CHIR99021) for 24 hrs. (**c,d**) Primary colon cancer cells HROC69 (passages 31–34) or HROC40 (p29–34) were treated with porcupine inhibitor LGK974 for 96–120 hrs or with 5 μM GSK3β inhibitor XVI (CHIR99021) for 24 hrs. **(e**) MEF Evi^Fl/Fl^ cells were transduced with a Cre-expressing construct and selected with hygromycin. 3 days after selection MEFs were collected for analyses of the indicated genes by qPCR. **(f)** MEF cells were treated with 5 μM GSK3β inhibitor XVI for 24 hrs. (**a–f**) After treatment cells were analyzed for expression of the indicated Evi/Wls target genes by qPCR. Relative data to control treatment were log_2_ transformed. Results of 3–6 independent experiments are shown as mean ± s.e.m. *p* values of every condition compared to control using Student’s *t*-test as well as values of independent experiments are shown in the Table S2.

Next, we investigated whether β-catenin-independent target genes were regulated in other colon cancer cells in a similar manner. In HT29 colon cancer cells, mRNA expression of *PLOD2, HADH* and *LCOR* as well as *AXIN2* as a canonical control target was downregulated upon treatment with the Porcupine inhibitor LGK974 (Fig. 3a). Moreover, treatment with the GSK3β inhibitor does not affect mRNA levels of *PLOD2, HADH* and *LCOR* but induced *AXIN2* mRNA expression (Fig. 3b). Down- or up-regulation of canonical Wnt signaling by targeting β-catenin/*CTNNB1* or APC with siRNAs did not change the levels of the non-canonical target genes in HT29 cells when *AXIN2* levels were regulated (Supplementary Fig. 4c). In DLD1 colon cancer cells *PLOD2* and *CLPTM1L* mRNA expression was downregulated after Evi/Wls silencing by shmirRNA (Supplementary Fig. 4b).

In addition, we analyzed expression of Wnt-dependent, β-catenin-independent target genes in primary colon cancer cells HROC69 and HROC40^55^. Similar to the results of colon cancer cell lines described above, we observed a downregulation of *PLOD2, HADH* and *LCOR* upon LGK974 treatment (Fig. 3c). Upon induction of canonical Wnt signaling by treatment with GSK3β inhibitor (CHIR99021) we observe changes in *AXIN2* mRNA level but not in *HADH*, *LCOR* or *PLOD2* mRNA levels in HROC69 and HROC40 cells (Fig. 3d, Supplementary Fig. 4d).

In order to investigate the gene regulation in a non-tumorigenic murine cell line we generated Evi/Wls-deficient mouse embryonic fibroblasts (MEF) by homologous recombination. The data confirmed a decrease in *Plod2, Hadh, Reep1, Clptm1l and Pros* expression in mouse cells (Fig. 3e). Treatment with GSK3β inhibitor IX (CHIR99021) for 24 hrs did not up-regulate *Plod2, Hadh, Clptm1l* and *Pros* mRNA expression in MEFs despite induction of *AXIN2* mRNA expression (Fig. 3f).

Taken together, these experiments confirmed *PLOD2, HADH*, and *LCOR* as non-canonical Wnt targets that depend on Wnt secretion but not on β-catenin in colon cancer cell lines as well as primary colon cancer cells and MEFs.

### Wnt5a/b is required for regulation of non-canonical Evi/Wls target genes

In order to identify Wnt ligands required for regulation of β-catenin-independent target gene expression in HCT116 cells we determined expression patterns of all 19 Wnt genes in these cells. Among others the non-canonical Wnt5b, Wnt11 and Wnt16, as well as the canonical ligands Wnt3, Wnt3a, Wnt9a and Wnt10b were detected by RNAseq experiments (Supplementary Fig. 5a). We confirmed presence of the non-canonical Wnt5b, Wnt11 and Wnt16 ligands in the supernatant of HCT116 cells by WB analysis (data not shown).

**Figure 4:**
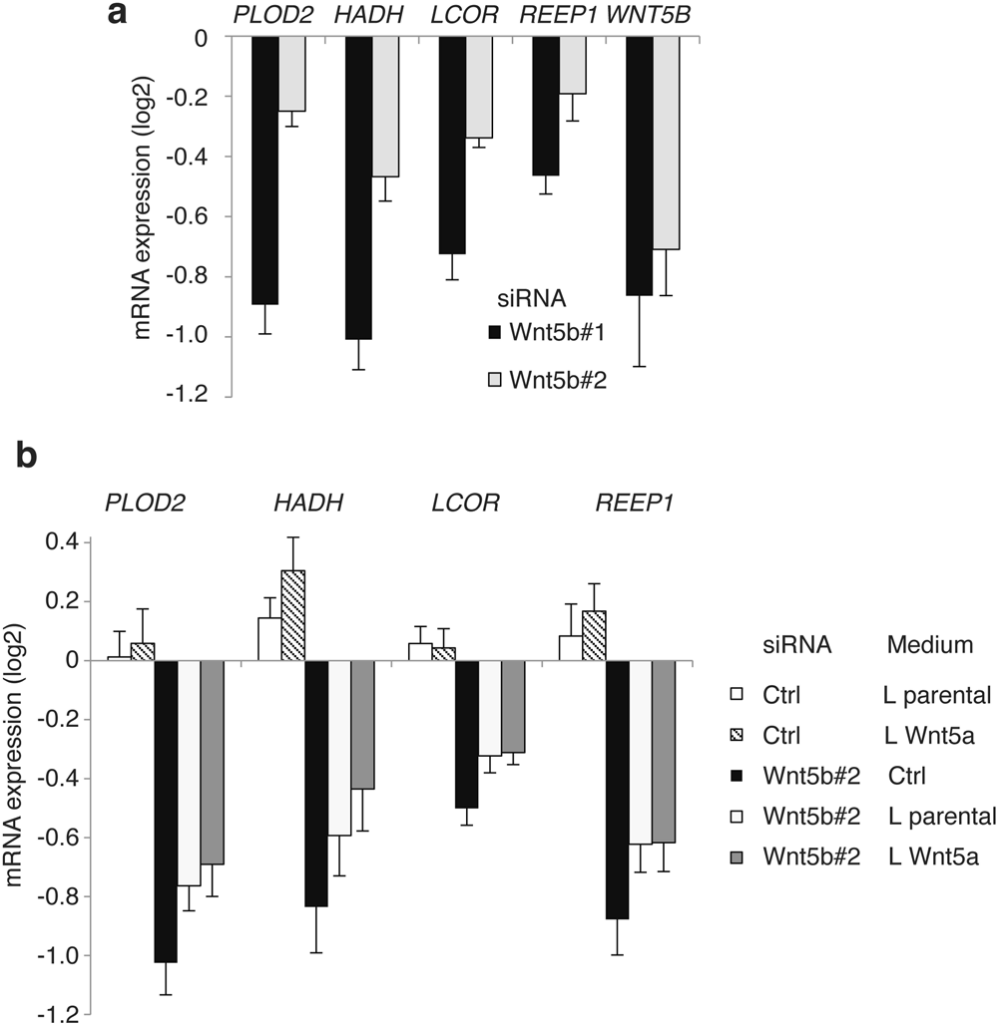
Wnt5a/b regulates identified Evi/Wls dependent non-canonical genes. (**a**) Silencing of Wnt5b in colon cancer cells leads to downregulation of Evi/Wls dependent target genes. HCT116 cells were reverse transfected with the indicated siRNAs and then analysis of the indicated genes by qPCR was performed. (**b**) Addition of Wnt5a partly rescues downregulation of non-canonical Wnt genes upon Wnt5b silencing. HCT116 cells were reverse transfected with siWnt5b#1 or siCtrl. 24 hrs later, medium from L cells (parental and mouseWnt5a) was added. Indicated non-canonical Wnt target genes were analyzed by qPCR. (**a,b**) Relative data to control treatments (siCtrl (**a**) or siCtrl Ctrl-medium (**b**)) were log_2_ transformed. Results of 4–8 independent experiments are shown as mean ± s.e.m. *p* values of every condition compared to control using Student’s *t*-test as well as values of independent experiments are shown in the Table S2.

Next, we silenced Wnt5b by siRNAs to investigate the transcriptional regulation of the predicted non-canonical target genes. Importantly, our results showed that depletion of Wnt5b downregulated mRNA expression of *HADH*, *LCOR, REEP1* and *PLOD2* (Fig. 4a). Addition of Wnt5a conditioned L-cell medium rescued the repression of non-canonical target genes after Wnt5b silencing (Fig. 4b). Conditioned medium from parental L-cells also had a weak effect on target gene expression, suggesting that these cells are not completely “Wnt-silent”. We indeed detected Wnt5a/b in the medium of parental L cells (Supplementary Fig. 5b).

Next, we tested whether we could rescue silencing Wnt5b-mediated downregulation of these genes by addition of recombinant Wnt3a (Supplementary Fig. 5c). In contrast to Wnt5a containing medium recombinant Wnt3a was not able to rescue mRNA expression of our target gene set. Then, we checked whether recombinant Wnt3a upon silencing of Evi/Wls has some impact on the expression of mRNA levels of *PLOD2, HADH*, *LCOR* and *REEP1* (Supplementary Fig. 5d). In line with Wnt5b silencing, recombinant Wnt3a was not able to rescue the downregulation of non-canonical targets, but it could rescue the downregulation of the canonical Wnt target gene *AXIN2*, which we used as positive control.

Taken together, our data demonstrated that Wnt5a/b controls expression of non-canonical, β-catenin-independent target genes in HCT116 cells. However, we cannot exclude that other Wnts might also influence expression of non-canonical target genes.

### Wnt5a/b is required for cancer cell proliferation

**Figure 5:**
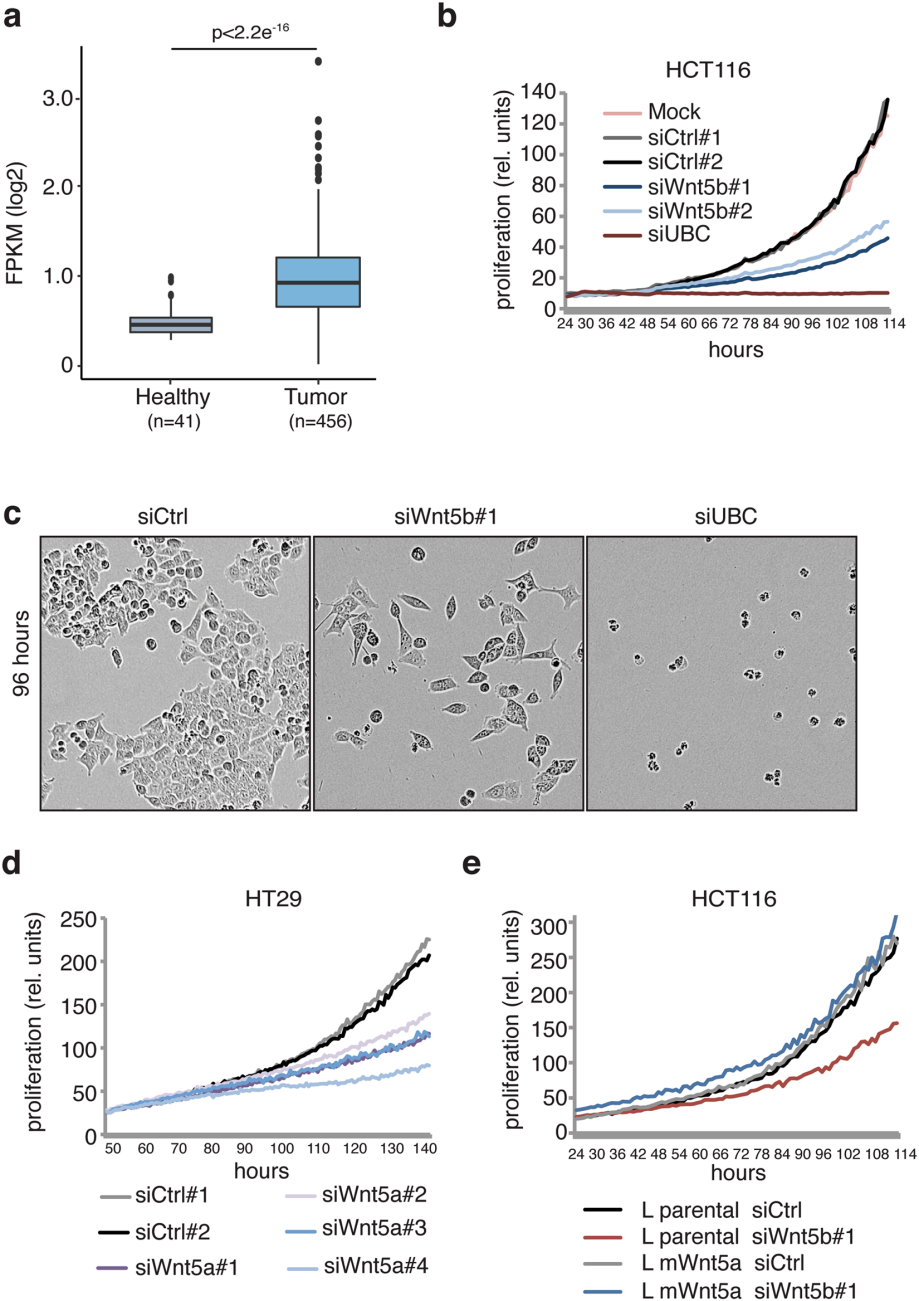
Wnt5a is overexpressed in colorectal cancer and influences the growth behavior of cancer cells. (**a**) *WNT5A* mRNA is overexpressed in colorectal cancer. RNAseq expression data (TCGA data set, 2017) were analyzed for expression of *WNT5A* in normal colon/rectum (healthy) *versus* colorectal cancer samples (tumor). n – number of cases used in the analysis. Significance of the differences in expression was calculated using the Student’s *t*-test. (**b–e**) Wnt5a/b is required for the survival of colon cancer cells. Cells were reverse transfected with the indicated siRNAs. In (**e**) 24 hrs later medium from L parental or Wnt5a cells was added. Then cells were monitored using the Incucyte Live Cell Imaging system and growth curves were plotted in relative units according to the manufacturer’s program. Representative experiments from 3 independent experiments are shown.

In order to investigate the role of Wnt5a/b in colon cancer, a TCGA colon cancer mRNA data set^56^ was used to compare Wnt expression levels in normal colon/rectum and adenocarcinoma tissue. This analysis showed that *WNT5a* mRNA expression is significantly upregulated (2.5-fold) in carcinoma compared to normal colon/rectum (Fig. 5a).

We next asked whether Wnt5a/b is required for proliferation of colon cancer cells. In long-term live cell analysis, silencing of Wnt5b with siRNAs led to impaired proliferation of HCT116 cells (Fig. 5b,c). These results suggest that Wnt5b has a growth-promoting role in HCT116 cells. A similar negative impact on cell proliferation after Wnt5a silencing was observed in HT29 colon cancer cells and in MDA-MB231 breast cancer cells (Fig. 5d, Supplementary Fig. 6a).

To exclude non-specific Wnt5b siRNA effects on cell proliferation, we performed an additional rescue experiments. Conditioned medium from Wnt5a-producing L cells was added to cells after silencing of Wnt5b with siRNAs. The negative effect on proliferation due to downregulation of Wnt5b was rescued, excluding off-target effects of siRNAs (Fig. 5e, Supplementary Fig. 6b). Together, these data suggest that proliferation of different types of cancer cell lines depends on Wnt5a/b.

### Wnt5a/b regulates proliferation *via* non-canonical signaling

To address the question whether Wnt5a/b regulates proliferation *via* canonical and/or non-canonical Wnt signaling, proliferation of HCT116 cells was analyzed after Wnt3/3a, Wnt5b and Evi/Wls silencing. Previously, we showed that proliferation of HCT116 cells was dependent on Evi/Wls expression and its depletion affected canonical Wnt signaling^51^. Depletion of Wnt3, Wnt3a or Wnt5b led to a similar decrease in cell proliferation as Evi/Wls silencing (Supplementary Fig. 7a). Thus, Wnt3/3a and Wnt5b might regulate proliferation *via* different signaling events.

Next, we addressed whether the proliferation defect caused by loss of Wnt5b was dependent on a canonical or a non-canonical signaling route. First, we analyzed whether proliferation could be rescued by inhibition of GSK3β, thereby activating β-catenin. GSK3β inhibition by application of the inhibitor CHIR99021 could not rescue HCT116 cell proliferation after Wnt5b silencing (Supplementary Fig. 7b). In contrast, proliferation after downregulation of Wnt3 and Wnt3a was rescued after drug addition (Supplementary Fig. 7b).

Taken together, our data indicate that Wnt5b promotes proliferation of colon cancer cells *via* non-canonical, β-catenin independent signaling. The proliferation defect upon Wnt5b downregulation could be rescued by addition of exogenous Wnt5a. These experiments suggest that both canonical and non-canonical ligands are required for proliferation of HCT116 cells.

### Non-canonical Wnt target genes are dependent on RoR2, Dvl2, ATF2 and ATF4

**Figure 6:**
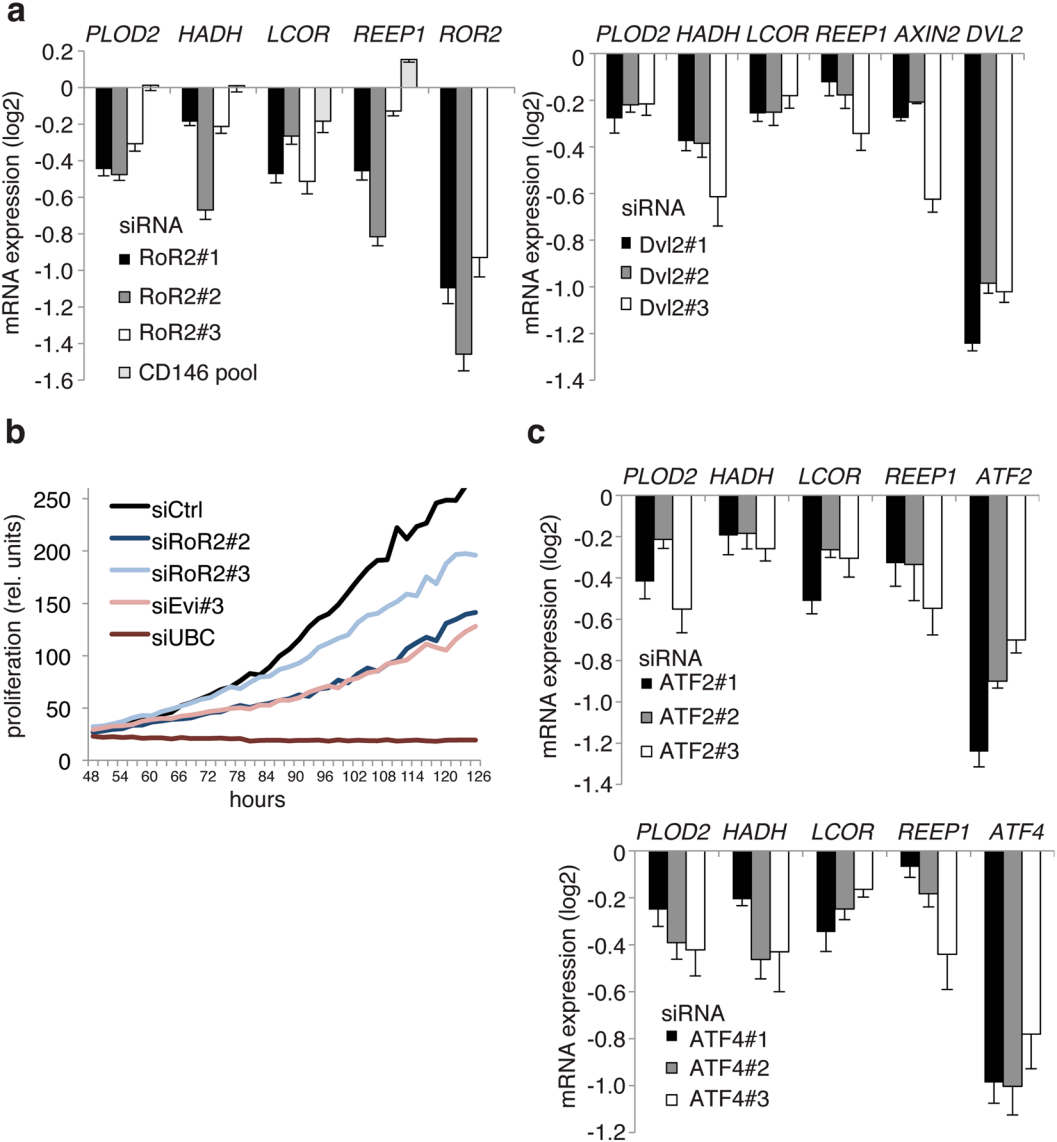
Evi/Wls regulated target genes are dependent on RoR2/Dvl2/ATF non-canonical Wnt pathway. (**a,c**) Silencing of RoR2, Dvl2, ATF2 and ATF4 leads to downregulation of the Wnt non-canonical target genes. HCT116 cells were reverse transfected with indicated siRNAs for 72 hrs and then expression of the indicated target genes was analyzed by qPCR. Relative data to siCtrl were log_2_ transformed. Results of 5–7 independent experiments are shown as mean ± s.e.m. *p* values of every condition compared to control using Student’s *t*-test as well as values of independent experiments are shown in the Table S2. (**b**) Silencing of RoR2 inhibits proliferation of HCT116 cells. Cells were reverse transfected with the indicated siRNAs. Then cells were monitored using the Incucyte Live Cell Imaging system and growth curves were plotted in relative units according to the manufacturer's program. Representative experiment from 3 independent ones is shown.

In contrast to the well-characterized canonical β-catenin Wnt signaling cascade, non-canonical Wnt signaling covers various cross-interacting signaling routes, which complicates analyses of these pathways. In order to test whether expression of the predicted non-canonical target genes, *PLOD2*, *HADH*, *LCOR*, *REEP1* were dependent on the RoR2/Dvl2 signaling, one of several known non-canonical Wnt pathways, we silenced RoR2/Dvl2 expression by siRNAs and subsequently analyzed the mRNA levels of target genes. As shown in Fig. 6a, knockdown of both RoR2 and Dvl2 reduced the expression of *PLOD2*, *HADH*, *LCOR*, *REEP1* indicating that this pathway is active in HCT116 cells (Fig. 6a).

Since we found that Wnt5b is important for proliferation of HCT116 colon cancer cells in β-catenin-independent manner we decided to test whether RoR2 is important for proliferation of these cells as well. In line with our Wnt5b results RoR2 silencing effects proliferation of HCT116 cells (Fig. 6b).

Previous studies indicated a cross-talk between JNK/AP-2/ATF2 and RoR2 non-canonical Wnt pathway^34,36,37,46,57,58^. In line, our promoter analysis revealed ATF2 as one of the enriched motifs in the non-canonical target gene cluster. As shown in Fig. 6c, silencing of ATF2 revealed downregulation of *PLOD2*, *HADH*, *LCOR* and *REEP1* mRNA expression; thus suggesting ATF2 as the transcription factor in non-canonical Wnt signaling in colon cancer cells. As the effect of silencing of ATF2 was not as pronounced as in the case of Evi/Wls or RoR2 downregulation we proposed that other homologous proteins could substitute it. Indeed, we identified ATF4 as another ATF family member that is potentially involved in non-canonical Wnt signaling, because *PLOD2*, *HADH*, *LCOR* and *REEP1* mRNA expression was comparably affected after ATF4 or ATF2 silencing (Fig. 6c).

### Non-canonical Wnt target gene expression correlates with Evi/Wls levels in colon cancer

**Figure 7:**
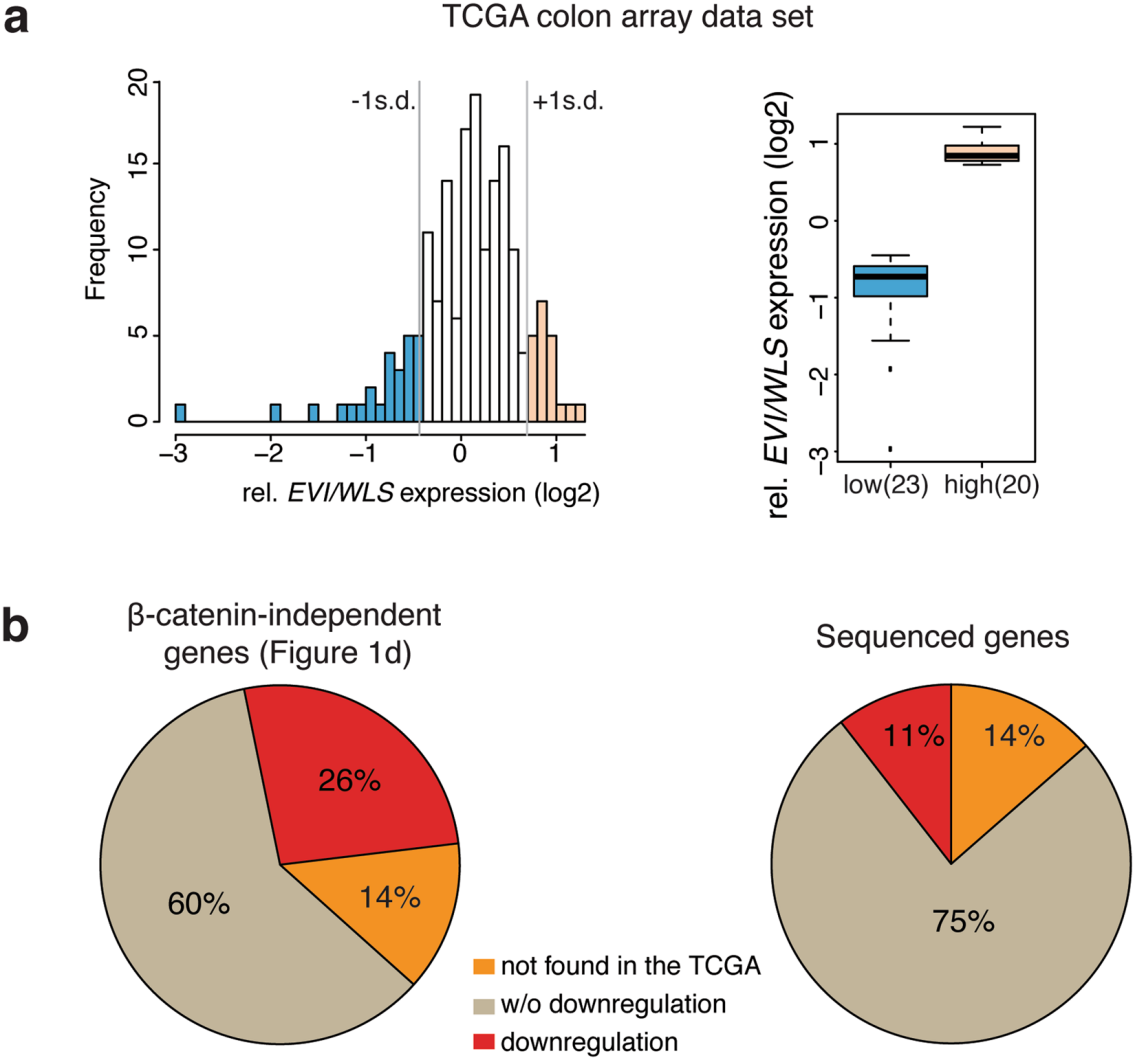
Wnt non-canonical target gene expression correlates with the mRNA level expression of Evi/Wls in human colon tumors. Microarray expression data of colorectal carcinoma was downloaded from the TCGA data portal. (**a**) Separation of microarray data in samples with low Evi/Wls expression and high Evi/Wls expression. Samples, which differ more than one standard deviation from the median Evi/Wls expression over all samples, were separated in samples with low Evi expression (<−1SD) and samples with high Evi expression (>+1 SD). 20 samples could be assigned to high Evi/Wls expression and 23 samples to low Evi/Wls expression. Median Evi expression between both groups has a fold change of 3 (*p*-Value: 1.0e^−13^). (**b**) Evi/Wls dependent Wnt non-canonical target genes correlate with Evi/Wls expression in human colon tumors. The percentage of identified Wnt non-canonical genes, which are significantly downregulated in the tumorsubset with lower expression of Evi/Wls, compared to the set of all genes, which were identified upon RNA sequencing of HCT116 cells (significance level *α* = 0.05; *p* = 3.9e^−8^, hypergeometric test).

The present study identified a set of genes, which are regulated by Evi/Wls but not by β-catenin canonical pathway. To look for evidence that Evi/Wls expression correlates with the expression levels of non-canonical Wnt target genes in tumors, we analyzed Evi/Wls expression levels in TCGA colon array data set^56^ (Oncomine, www.oncomine.org, TCGA) (Fig. 7a). Samples with low or high Evi expression were separated into two groups to distinguish two patient cohorts. Comparing the expression levels of non-canonical target genes from our cluster analysis (Fig. 1d, Supplementary Table 1) Evi/Wls low *versus* high expressing groups revealed that 26% of these genes were also significantly downregulated (*p* = 3.9e^−8^, hypergeometric enrichment test) in the Evi/Wls deficient cohort (Fig. 7b, Supplementary Table 3). These data suggest that the mRNA expression levels of the identified non-canonical genes correlate with mRNA expression levels of Evi/Wls in colon cancer samples. This set of genes can be used for identification of unknown regulators of non-canonical Wnt signaling regulators and identification of non-canonical Wnt signatures.

## Discussion

Non-canonical Wnt signaling and its role in tumorigenesis still remains poorly understood. The signaling routes downstream of the receptors are complex; there is still a lack of cellular markers to assess the activity of this pathway. For canonical, β-catenin-dependent signaling, the identification of *AXIN2*^59^, *CCND1/cyclin D1*^60^, *BRACHYURY*^61^, *PITX2*^61^ and *MYC*^62^ greatly accelerated the analysis of Wnt pathways during development and disease. To date, comparable target gene sets for non-canonical Wnt signaling are not known and their discovery opens new possibilities to investigate non-canonical signaling. Additionally, new non-canonical target genes might facilitate the identification of small molecule antagonists and agonists in β-catenin-independent Wnt signaling.

In this study, we used a perturbation and profiling approach to identify target genes that are directly or indirectly controlled by non-canonical, β-catenin-independent Wnt signaling in colon cancer cells. We used HCT116 cells as a model system that expressed endogenous canonical and non-canonical Wnt ligands to perform perturbations inhibiting either the secretion of Wnts or β-catenin signaling followed by expression profiling. Using an iterative signature algorithm, we identified several candidate genes that are dependent on autocrine, non-canonical Wnt signaling and validated their expression response in multiple independent cell models using both genetic and pharmacological approaches. Computational analysis revealed that several of the predicted non-canonical Wnt target genes contain ATF2 binding motifs in their promoter regions. In line with this, we showed that ATF2 as well as ATF4 led to downregulation of identified non-canonical target genes, including *PLOD2*, *HADH, LCOR* and *REEP1*. Our experiments showed that these target genes also depend on Dvl2 and RoR2 which is in line with current knowledge of the role of RoR2/Dvl2 in non-canonical Wnt signaling^37–39^.

Other canonical but non-transcriptional pathways induced by Wnt ligands were recently discovered. For example, Wnt/STOP signaling stabilizes GSK3β-target proteins^63,64^, which are required for multiple biological processes. Stabilization of GSK3β-target proteins could lead to a secondary effect on our identified target genes, as they are independent of β-catenin-mediated transcription. However, we found that GSK3β-inhibition did not affect the predicted non-canonical target genes thereby excluding Wnt-STOP dependent regulation.

The role of Wnt5a in tumorigenesis is tissue specific. In melanoma, Wnt5a correlates with tumor progression and has been shown to play an important role in epithelial mesenchymal transition (EMT) and cell migration^65,66^. Wnt5a expression has also been shown to enhance resistance to BRAF inhibitors^35^. Recently, it was shown that in breast cancer Wnt5a inhibits proliferation of tumour-initiating cells *via* TGFβ/SMAD signaling. Furthermore, heterozygous loss of *WNT5A* correlated with shorter survival of breast cancer patients^67^. In addition, it was recently shown Wnt5a ability to promote breast cancer progression is also dependent on the cellular context^68^. In colon cancer, the current model suggests that Wnt5a inhibits canonical β-catenin dependent Wnt signaling *via* RoRα^69^. However, we showed that proliferation of colon cancer cells depends on Wnt5a/b independent from the canonical Wnt pathway. We further showed that Wnt5a/b as non-canonical ligands were responsible for regulation of these target genes in colon cancer cells. Although we identified Wnt5b as the main regulator of non-canonical Wnt target genes in HCT116 cells, we cannot exclude that other non-canonical Wnt ligands can substitute Wnt5a/b in other cellular systems.

Since our experiments are based on low, endogenous levels of Wnt5a/b, we cannot exclude that high expression of Wnt5a/b could have an opposite effect. When Wnt5a is overexpressed, it can substitute canonical Wnt proteins by blocking their FZDs. The requirement of Wnt5a for proliferation also correlates with an upregulation of Wnt5a as shown by TCGA. This result is also consistent with observations made by Yoshida, N. *et al*.^70^ and Gujral, T. S. *et al*.^71^, which showed that Wnt5a is overexpressed in colon cancer.

In conclusion, we demonstrate how a systematic approach can be applied for the identification of target gene clusters, which expression is dependent on specific downstream signaling routes. Iterative signature algorithm predicted specific non-canonical, β-catenin independent target genes. Several of them were validated in different cellular models. Future studies on Wnt non-canonical pathway regulators and their interplay with the canonical pathway will be necessary to improve our knowledge on Wnt signaling in colon cancer. Our data may open new methods for the analysis of non-canonical Wnt signaling in cancer cells.

## Material and Methods

### Cell Culture

All cell lines, except MEFs, were obtained from ATCC. HCT116, HT29 (McCoy’s; GIBCO), L cells, HEK293T, MDA-MB231, MEF (Dulbecco’s MEM; GIBCO), DLD1 (RPMI; GIBCO) cells were cultured without antibiotics unless they were stably transduced. Media were supplemented with 10% fetal bovine serum (PAA). Primary colon cancer cell lines HROC40 and HROC69 were used on passage 28–34 and treated with 20 uM LGK974 for 120–144 h before analysis of Evi/Wls target gene expression. Cell lines were culture in DMEM supplemented with 20% fetal bovine serum (PAA). All cells were regularly tested for the mycoplasma contamination.

### Quantitative PCR (qPCR)

cDNA was prepared from 0.5–1 µg total RNA, using the RevertAid H Minus First Strand cDNA Synthesis Kit (Thermo Fischer Scientific) according to the manufacturer’s protocol (Thermo Fischer Scientific). cDNA was diluted to 5ng/µl and used for qPCR on the Lightcycler480 (Roche), using the universal probe library system (Roche) in a 384-well format. UBC was used as a reference gene for relative quantification. Oligonucleotide sequences are listed in Supplementary Table 4.

### siRNA transfection

siRNAs were obtained from GE Dharmacon (GE Healthcare) or Ambion (Thermo Fischer Scientific). Cells were transfected using a total of 0.1% Lipofectamine RNAiMAX Transfection Reagent (Thermo Fischer Scientific). Sequences of siRNAs used in this paper are shown in Supplementary Table 5.

### Biclustering with the Iterative Signature Algorithm (ISA)

The normalized RNAseq data of siRNA knockdowns (siCTNNB, siAPC, siEvi) and normal state (Ctrl) in HCT116 cells are provided in Moffa *et al*.^49^. The data set is available at the ArrayExpress database with accession number E-MTAB-651 (https://www.ebi.ac.uk/arrayexpress/experiments/E-MTAB-651).

The eisa package (v 1.12.0), part of the Bioconductor package suite, was employed to perform the biclustering. First, the RNAseq data was combined to a matrix, where the columns represent the conditions (siRNA knockdowns) and the rows the genes. Subsequently, genes were filtered with a variance greater than 0.5 and expression higher than 3 RPKM in at least 2 conditions. In the next step two normalized matrices were generated. Gene expression values were normalized to an average of 0 and a variance of 1, in one matrix across the columns (conditions), in the other across the rows (genes). The algorithm uses two user-defined thresholds, t_c_ and t_g_, which determine how closely related are the genes (corresponds to t_g_) or conditions (corresponds to t_c_) within the transcription module. We used t_c_ = 0.45 and t_g_ = 1.75. Before starting the algorithm an initial set of genes has to be provided. The ISA will stepwise refine this input by finding the driving conditions for these genes and including/removing genes that are well/poorly co-expressed with the input set. The iterative manner of the algorithm will find a set of co-regulated genes by its self-consistency. Therefore we used 300 randomly generated gene sets as input and filtered duplicated outputs with a total gain of 23 unique transcription modules^72,73^. The script can be accessed at https://github.com/boutroslab/Supplemental-Material.

### Analysis of TCGA data for WNT5A mRNA expression

The data from TCGA^56,74^ colorectal carcinoma data set TCGA-COAD (for Colon Adenocarcinoma; 497 samples). Samples were divided into tumor samples and healthy samples according to their barcode as described in the TCGA Wiki (https://wiki.nci.nih.gov/display/TCGA/TCGA + barcode; 09/25/2017). FPKM-normalized RNA sequence samples were log2-scaled. Statistical significance of gene expression differences between healthy tissue samples and tumor samples was determined using a two-sample Student’s *t*-test.

### Differential gene expression of high and low expressed *EVI/WLS* mRNA samples

Samples with a greater/lower expression than at least one standard deviation (SD) from the median *EVI/WLS* mRNA expression over all samples were defined as high/low *EVI/WLS* expression. Differential gene expression between those samples was calculated with the multtest package (v 2.16.0) part of the Bioconductor suit. *t*-test was applied to determine *p*-values, which were corrected for multiple testing (FDR, Benjamini-Hochberg)^75^. The script can be accessed at https://github.com/boutroslab/Supplemental-Material.

## Electronic supplementary material

Supplementary information.

Table S1

Table S2

Table S3
