# Supplementary material for: β-catenin-independent regulation of Wnt target genes by RoR2 and ATF2/ATF4 in colon cancer cells
Source: Sci Rep. 2018 Feb 16;8:3178. doi: 10.1038/s41598-018-20641-5 (PMC5816634; doi:10.1038/s41598-018-20641-5)
Supplement: Supplementary file 1 — Supplementary information. [file 41598_2018_20641_MOESM1_ESM.pdf]

β-catenin-independent regulation of Wnt target genes by RoR2 and ATF2/ATF4 in colon cancer cells

Oksana Voloshanenko, Uwe Schwartz, Dominique Kranz, Benedikt Rauscher, Michael Linnebacher, Iris Augustin, Michael Boutros

Supplementary information:

Supplementary Figures 1-7

Supplementary tables 4-6

R code - ISA_clustering

R code - TCGA_data_analysis

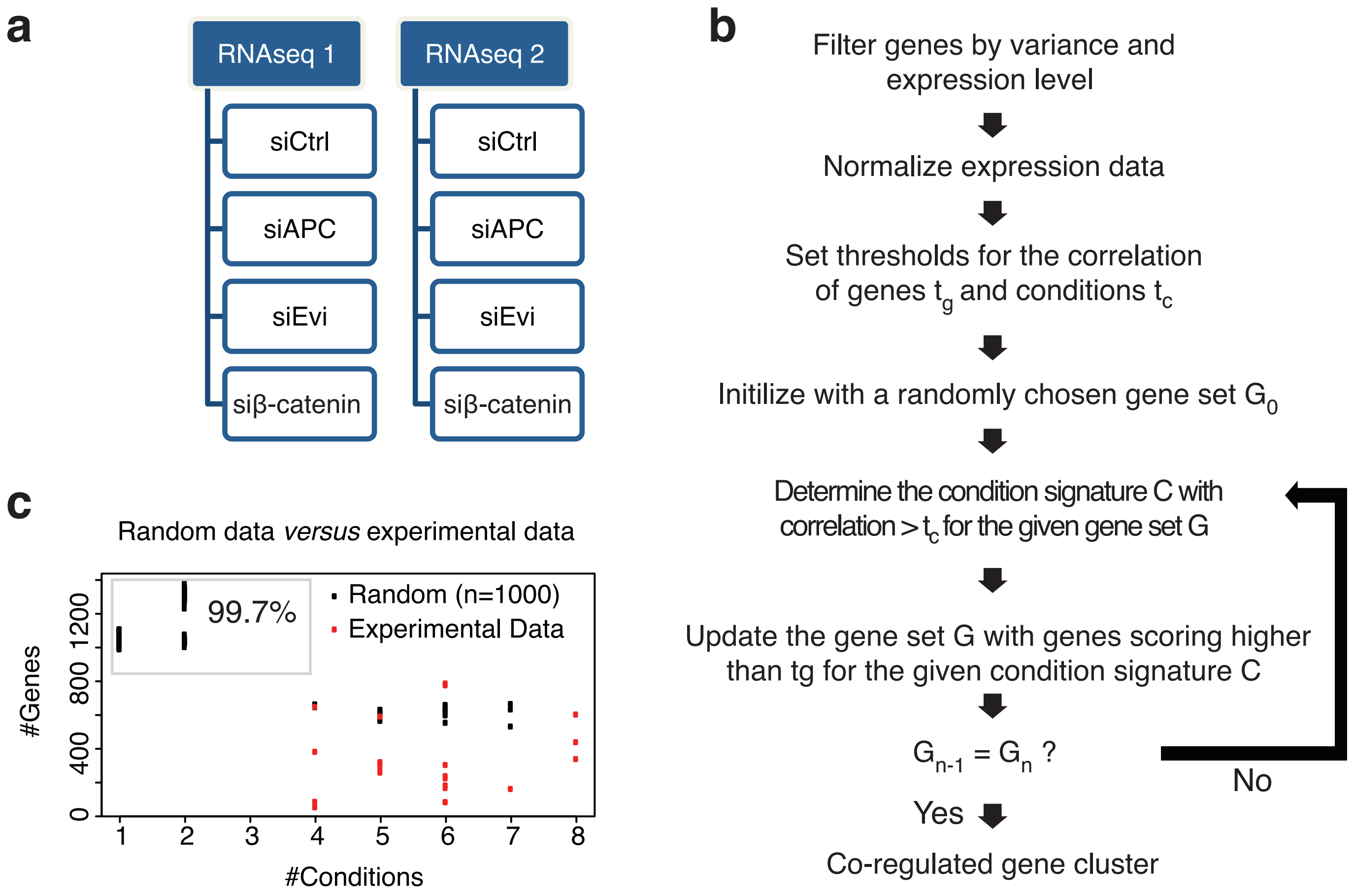

Supplementary Figure 1. Distribution of clusters upon analysis of random data set *versus* experimental data. (**a**) RNAi RNAseq conditions which were used in cluster analysis. (**b**) The detailed steps of bioinformatical analyses for identification of meaningful biological clusters on the basis of RNAi RNAseq HCT116 data. (**c**) Clusters derived from randomized data consisted of large gene sets correlated across a few conditions. In contrast, clusters derived from the experimental data exhibited a distinct pattern with small gene sets co-regulated across several conditions. Each dot on the graphic represents one cluster. 99.7% of all clusters generated from randomized data consisted of less than 2 conditions and more than 900 genes.

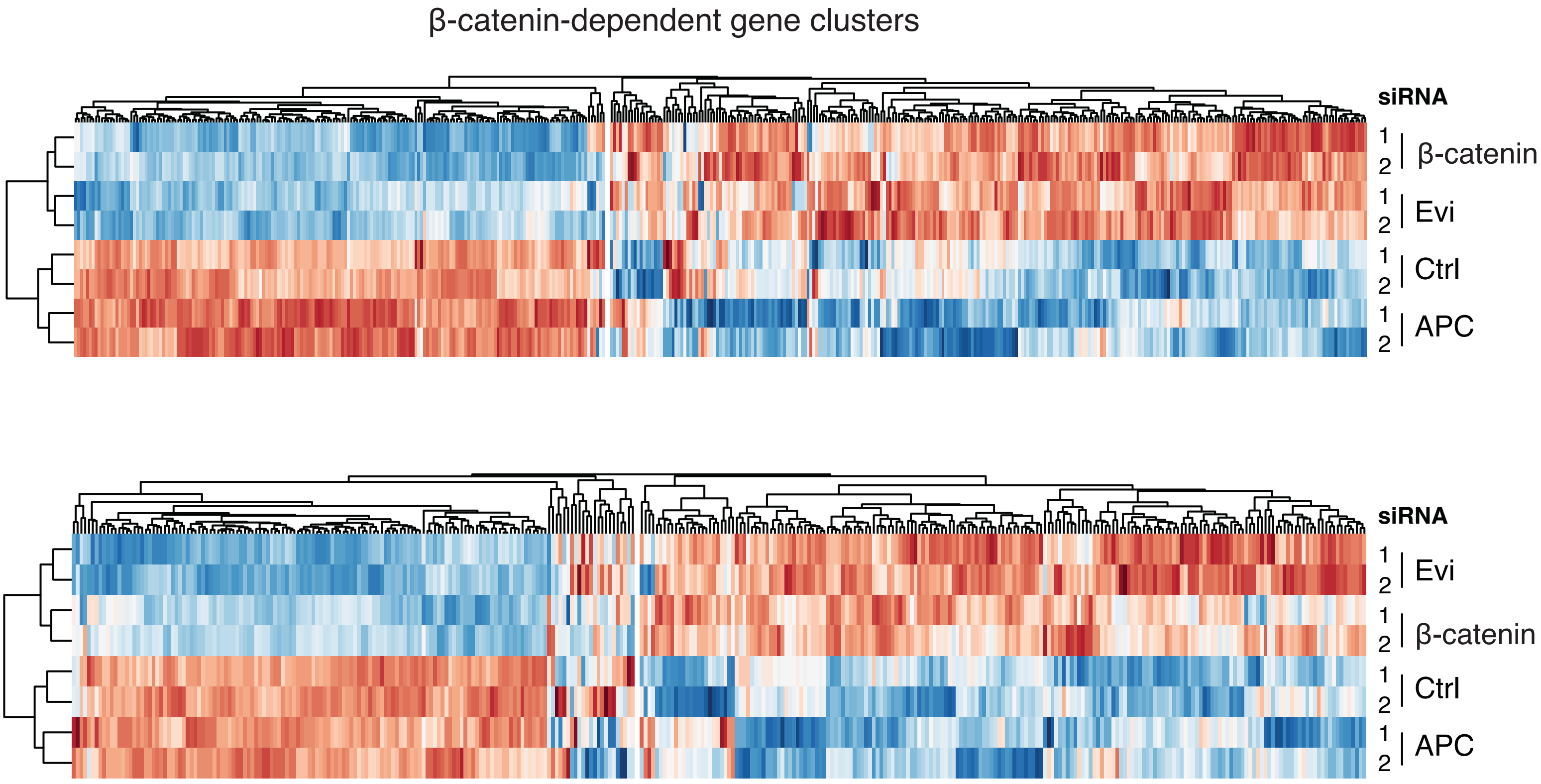

Supplementary Figure 2. β-catenin dependent Evi/Wls-regulated genes clusters generated by ISA (Iterative Signature Algorithm), which include all 4 conditions.

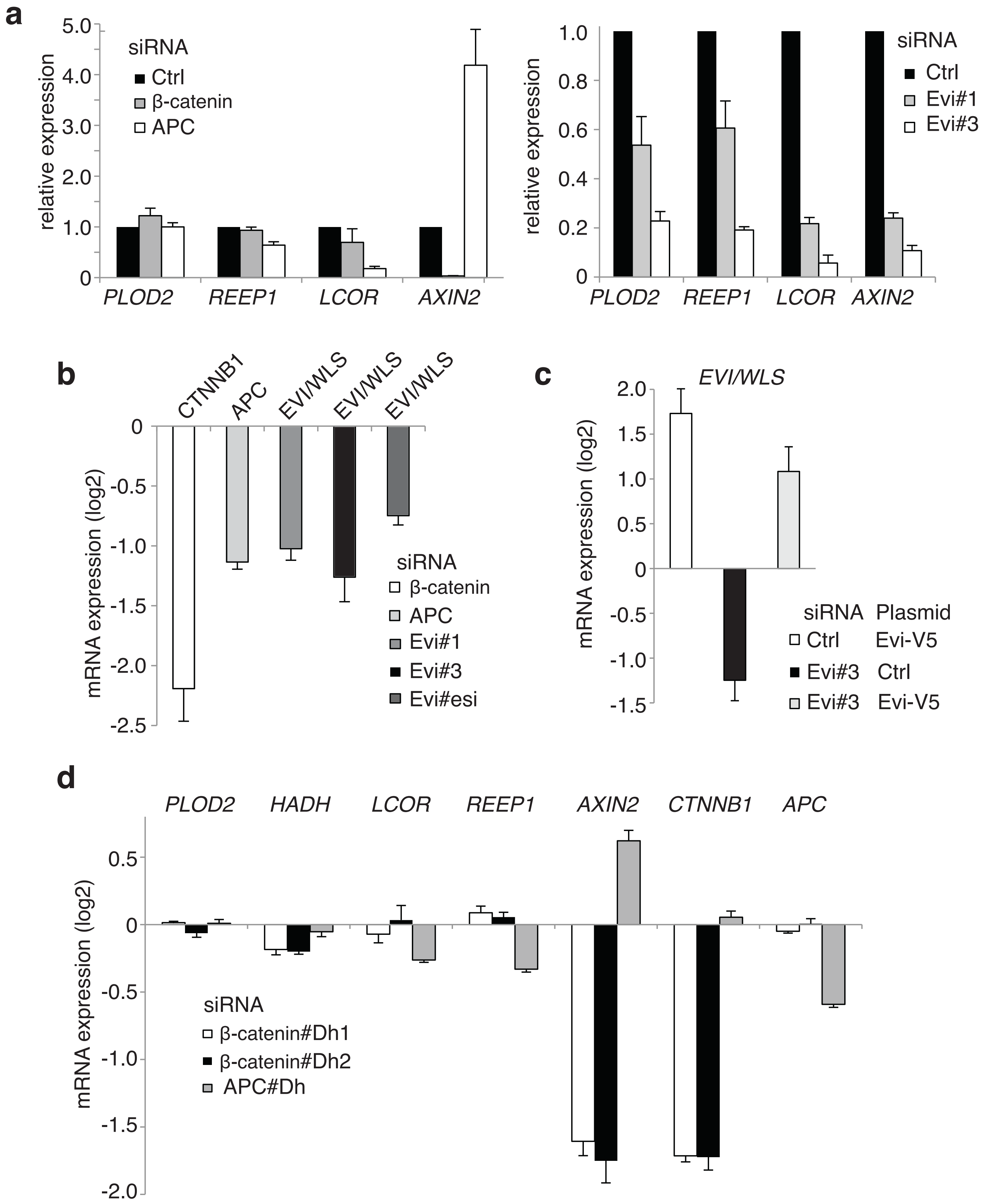

Supplementary Figure 3. Wnt non-canonical genes are regulated by Evi/Wls but not *via* the canonical Wnt pathway. (a-d) HCT116 were reverse transfected with indicated siRNAs for 72 hrs and gene expression was analyzed by qPCR. (a) Relative mRNA expression shown without log2 transformation and normalized to siCtrl (1) for Figure 2a. (b,c) Relative expression is shown for Figures 2a (b), Figure 2c (c). (d) Experiment similar to Figure 2a with different siRNAs. (a-d) Results of 3-6 independent experiments are shown as mean ± s.e.m.

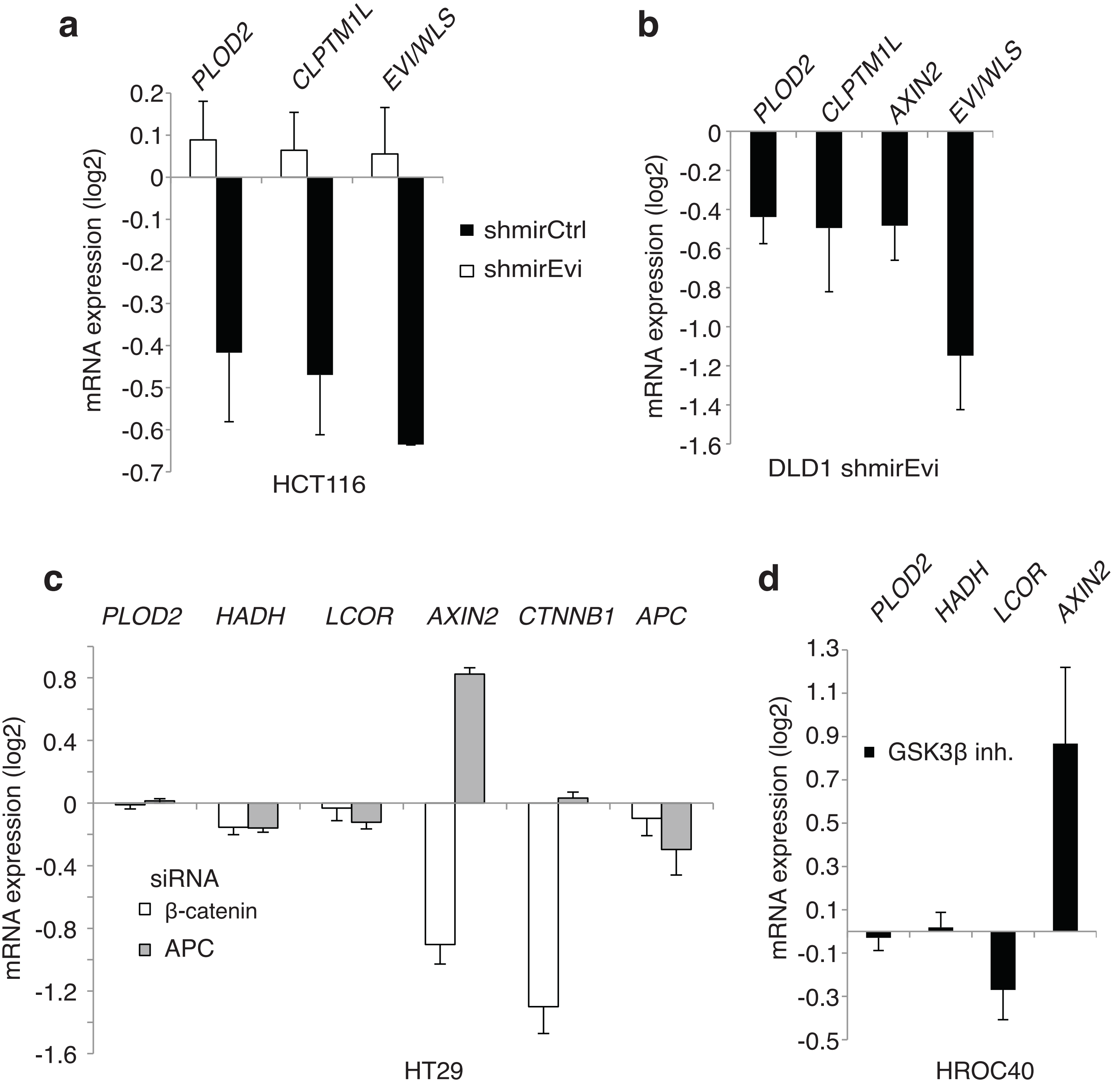

Supplementary Figure 4. Downregulation of Evi/Wls in colon cancer cells regulates of non-canonical target genes. a,b) Inducible silencing of Evi/Wls with shmirRNA in HCT116 and DLD1 cells leads to downregulation of non-canonical Wnt target genes. HCT116 or DLD1 cells were transduced with a doxcycline-inducible shmirEvi or shmirCtrl constructs and treated with or without doxcycline for 72(HCT116) or 96 (DLD1) hours. (**c**) HT29 colon cancer cells were reverse trasnfected with β-catenin or APC siRNAs for 72 hrs. (**d**) HROC40 colon cancer cells (p29-34) were treated with 5 µM GSK3β inhibitor XVI (CHIR99021) for 24 hrs. (a,b,c, d) After the indicated treatments cells were analyzed for expression of the Evi/Wls regulated target genes by qPCR. Results of 3 independent experiments are shown as mean ± s.e.m.

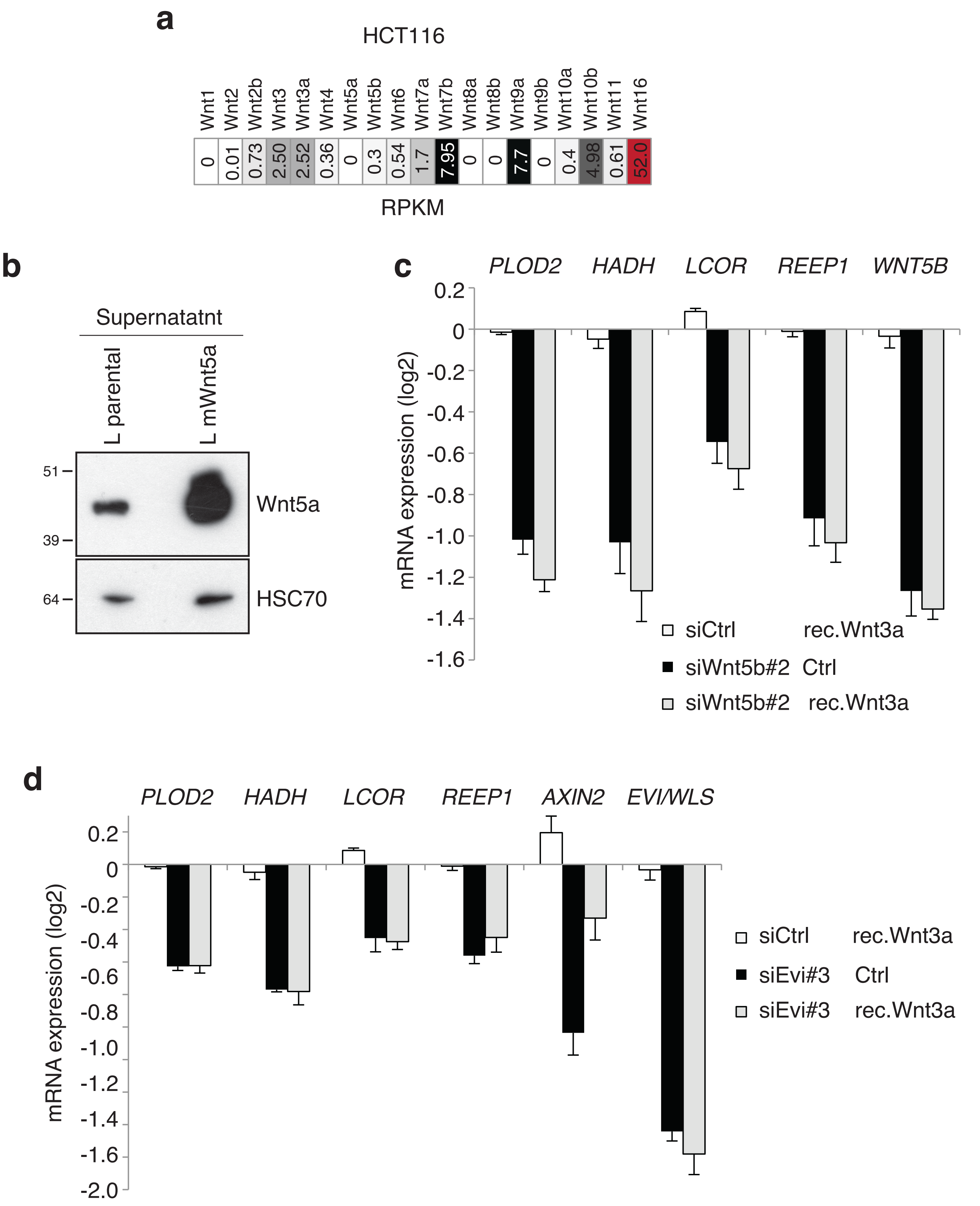

Supplementary Figure 5. Downregulated non-canonical target genes upon silencing of Wnt5b or Evi/Wls are not rescued by addition of recombinant Wnt3a. (a) RNA Wnt expression in colon cancer HCT116 cells. Average of RPKM values of all conditions shown in Figure 1 c and d. **(b)** Parental L cells secrete basic amount of Wnt5a. Medium from mouse L parental and Wnt5a cells was collected. Wnts in the media were enriched by Blue sepharose pulldown and loaded on the gel for WB detection. (**c,d**) HCT116 colon cancer cells were reverse transfected with the indicated siRNAs. 24 hrs later 200 ng/ml of recombinant mouse Wnt3a was added for next 48 hrs and then the non-canonical target genes were analyzed by RT-qPCR. Results of 3 independent experiments are shown as mean ± s.e.m.

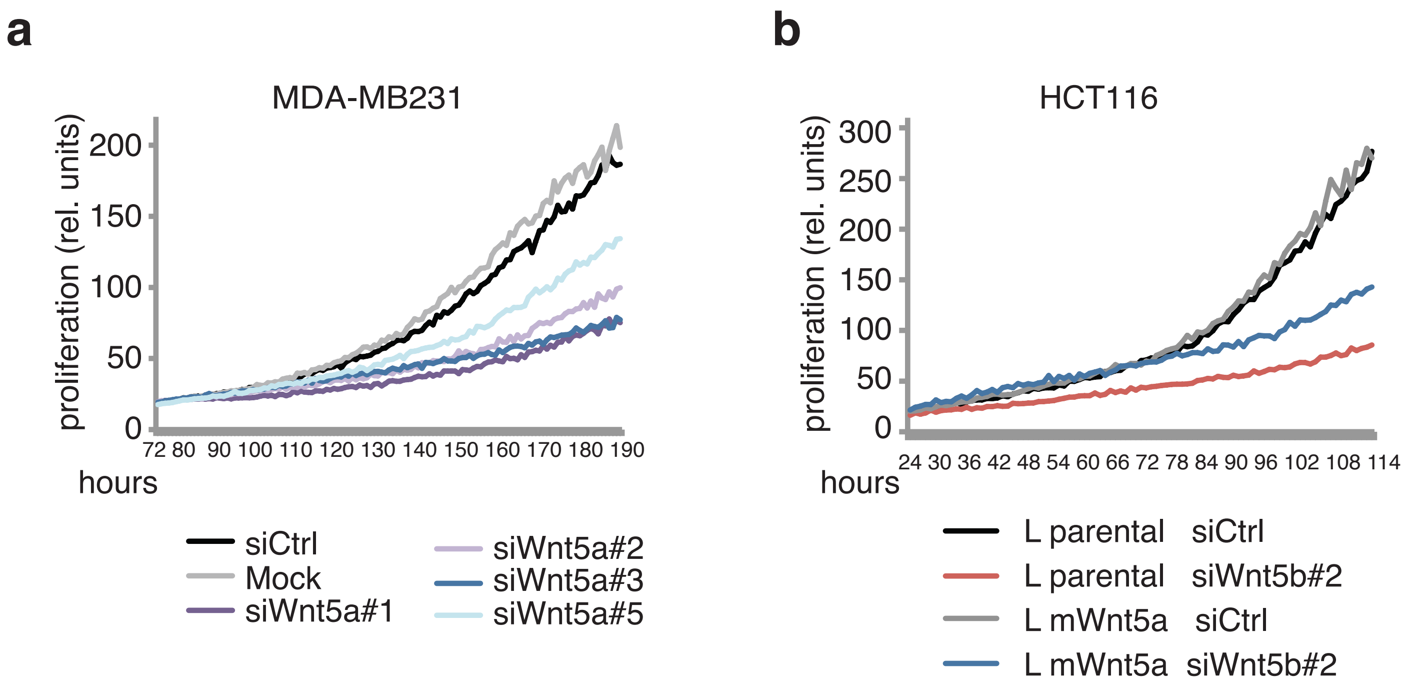

Supplementary Figure 6. Wnt5a is required for survival of cancer cells . (a,b) Cells were reverse transfected with the indicated siRNAs and then monitored using the Incucyte Live Cell Imaging system. Growth curves were plotted in relative units according to the manufacture program. Representative experiment from 3 independent experiments is shown. (b) 24 hrs after siRNA transfection HCT116 cells were treated with the indicated medium.

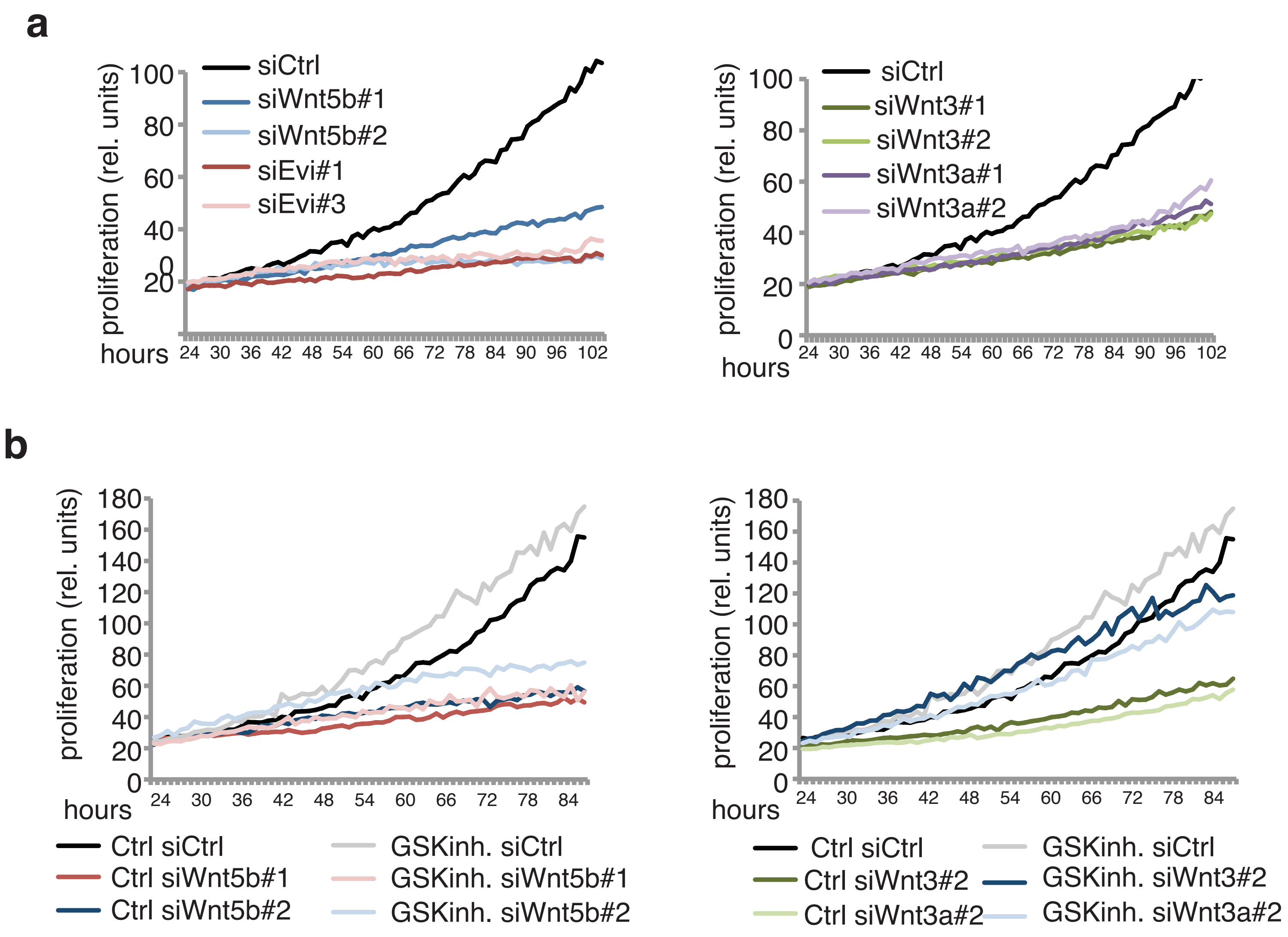

Supplementary Figure 7. Inhibition of cell growth upon downregulation of Wnt5b can be rescued by addition of endogenous Wnt5a independent from induction of canonical Wnt pathway. (a) Addition of mouse Wnt5a rescues phenotype induced by downregulation of Wnt5b. (**b**) Downregulation of Wnt5b inhibits growth of cells similar to silencing of Evi/Wls and Wnt3/3a. (Left and right panels from the same experiment. Ctrl data are the same in both panels). (**c**) Cell growth inhibition upon silencing of Wnt5b in HCT116 cells cannot be rescued by induction of canonical Wnt pathway using GSK3β inhibitor. (a,b,c) Cell growth behaviour of HCT116 cells was monitored using the Incucyte Live Cell Imaging system after reverse siRNA transfection. GSK3β inhibitor XVI CHIR99021 or medium from L parental/mWnt5a cells were added 24 hrs after siRNA transfection. Representative experiments from 3 independent experiments are shown.

Supplementary Table 4 - qPCR primers

Gene	Forward Primer	Reverse Primer	UPL Probe
HUMAN			
APC	GCATGGACCAGGACAAAAAT	GAACACACACAGCAGGACAGAT	17
WLS/EVI	TCATGGTATTTCAGGTGTTTCG	GCATGAGGAACTTGAACCTAAAA	38
AXIN2	AGAGCAGCTCAGCAAAAAGG	CCTTCATACATCGGGAGCAC	88
UBC	GGCAAAGATCCAAGATAAGGAA	GGACCAAGTGCAGAGTGGAC	11
CTNNB1	GCTTTCAGTTGAGCTGACCA	CAAGTCCAAGATCAGCAGTCTC	21
DVL2	TATTTCACTCTCCCCCGAAA	GGAAGGTGCCAGTCAGAGC	27
PLOD2	ATGGAAATGGACCCACCA	TGCAGCCATTATCCTGTGTC	2
REEP1	CACAGACATCTTCCTTTGTTGG	CTGGAGCCTTTTGTGTAGGG	71
CLPTM1L	CCGGGGAGTCTGATACACA	GTTGTCCGCCATCACGTT	28
LCOR	GTTGTCCGCCATCACGTT	ACCACTCCGAAGTCCGTCT	14
HADH	CTCGGCCAAGAAGATAATCG	TCTACCAACACTACTGTGTGACCA	61
ROR2	CCCGATTCCAACTCTGAAAG	GGCCTTGGACAATGGTGAT	36
ATF2	TCCAGCTGCAGTCCCACT	TTTTTGCTTCTGACTGTACTGGTT	55
ATF4	TCTCCAGCGACAAGGCTAAG	CCAATCTGTCCCGGAGAA	76
MOUSE			
WLS/EVI	CCCAGCCATGAGCAAAGT	GCATGAGGAACTTGAACCTGA	75
REEP1	GGTGATGGTGCTCCTGCT	GGGATCGTAGGGTTCTAGGC	22
PROS	CAAGGAAATAATTGAAGGAAAACAA	GCCAACCCTCTGCATTAGTT	38
PLOD2	ACATTGGGAAACGCTACCTG	CATTATCCTGCAGATTCCACTG	93
CLPTM1L	CTTCCTGCTGGATGAACAGAC	CCTTCTTCACTTTCCAAAGCTC	40
HADH	TCCTGATCAAGCATGTGACC	CACCAATACTACTGTATGGCCAGTT	70
LCOR	TGCTCCAGTACTCAAGGGAAC	TGGTTCCATCCTGTAAGCTTCT	9
UBC	GTCTGCTGTGTGAGGACTGC	CCTCCAGGGTGATGGTCTTA	77
SDHA	TGTTCAGTTCCACCCCACA	TCTCCACGACACCCTTCTGT	71

Supplementary Table 5 - siRNAs

**Target gene symbol**	**siRNA ID Ambion(s)/Dharmacon(MU)**	**Sequence – sense strand**
Ctrl/control*	*Silencer*® Select Negative Control #1 and 2	
APC*	s1433	GGAUCUGUAUCAAGCCGUUtt
β-catenin/CTNNB1*	s438	CUGUUGGAUUGAUUCGAAAtt
Evi#1*	s36745	GGACAUUGCCUUCAAGCUAtt
Evi#3	s36747	GGAUUUCCAUGACCUUUAUtt
Evi#esi	Sigma EHU088941	Endoribonuclease-prepared siRNAs are pools of siRNAs resulting from cleavage of long double-stranded RNA (dsRNA) with *Escherichia coli* RNase III.
UBC	s14559	GUGAAGACCCUGACUGGUAtt
Wnt3a#1	s195523	GGAAGGUUCCAUGAAGCGAtt
Wnt3a#2	s195524	GCCAUGAACCGCCACAACAtt
Wnt3#1	s14868	CGAUAUCCUGGACCACAUtt
Wnt3#2	s14869	GCAAUUACAUCGAGAUCAUtt
Dvl2	MU-004069-01	GACAGAAACCGAGUCAGUA<br>UGUGAGAGCUACCUAGUCA<br>CGCUAAACAUGGAGAAGUA
Wnt5b	MU-004069-01	GCAGGGCUGUGUAUAAGAU<br>AGAGGAAGCUGUGCCAAUU
Wnt5a	MU-003939-01	GUUCAGAUGUCAGAAGUAU<br>UCAGAUGUCAGAAGUAUAU<br>GCGACAACAUCGACUAUGG<br>GGUCGCUAGGUAUGAAUAA
Wnt5a#5	s14872	AGAUGUCAGAAGUAUAUtt
ATF2	MU-009871-02	GAAGAAAUCUGGCUAUCAU<br>GACAAACCCUUUCUAUGUA<br>GAAGUGGGUUUGUUUAAUG
ATF4	MU-005125-02	GAUCAUUCCUUUAGUUUAG<br>CAUGAUCCCUCAGUGCAUA<br>GUUUAGAGCUGGGCAGUGA

RoR2	MU-003172-01-0002	GGAACTCGCTGCTGCCTAT<br>GCAGGTGCCTCCTCAGATG<br>GCAATGTGCTAGTGTACGA<br>GAAGACAGAATATGGTTCA
β-catenin#Dh	D-003482-03/04	CCACTAATGTCCAGCGTTT<br>ACAAGTAGCTGATATTGAT
APC#Dh	D-003869-05	GAAATAGGATGTAATCAGA

* siRNAs used in the RNAsequencing experiment.

Table S6 - Constructs

Short name	Name	Source
lentiviral packaging	pMDM2-VSVG; psPAX2	
pLenti Evi-V5	pLenti-hWls/Evi V5-His	Re-cloned from the plasmid provided by X. Lin[1]
shmirCtrl	pTRIPZ shmirCtrl	Thermo Scientific
shmirEvi	pTRIPZ shmirEvi (GGCGTCACAGTCCAAGTGA)	Thermo Scientific
Cre-recombinase	pLenti_Cre-recombinase_Hygro	provided by J. Silke[2]

1 Belenkaya, T. Y. *et al*. The retromer complex influences Wnt secretion by recycling Wntless from endosomes to the trans-Golgi network. *Dev Cell* **14**, 120-131, doi:10.1016/j.devcel.2007.12.003 (2008).

2 Vince, J. E. *et al*. IAP antagonists target cIAP1 to induce TNF alpha- dependent apoptosis. *Cell* **131**, 682-693, doi:10.1016/j.cell.2007.10.037 (2007).

ISA Bi-Clustering

Uwe Schwartz

13 8 2017

Dependencies

```
library(eisa)
library(Biobase)
library(genefilter)
library(org.Hs.eg.db)
```

Read RPKM-count tables

First we load the RPKM-count tables obtained from Moffa et al. In this study RNA-expression levels were quantified after single gene knock-down of WNT-signaling pathway components in HCT116 cells. Each table represents one biological replicate. In this study our focus lies on APC, CTNNB1 and EVI knock-downs and the control sample.

```
rna1<-read.table("data/RNA-seq/SA00011.seq0005.rpkm", header=T, row.names=1)
rna2<-read.table("data/RNA-seq/SA00011.seq0006.rpkm", header=T, row.names=1)

# mark second replicate
colnames(rna2)<- paste(colnames(rna2), "2", sep="_")
# select samples of interest and convert them to an ExpressionSet object
rnaseq<-cbind(rna1[,c(1,4,5,6)],rna2[,c(1,4,5,6)])
ma.rna<-as.matrix(rnaseq)
exp.set<-new("ExpressionSet", exprs=ma.rna, annotation="org.Hs.eg")
```

Non-specific filtering

Next, we remove not expressed genes and genes exhibiting a low variability across all samples.

```
# gene has at least in 2 samples RPKM-values >3
kLimit<-2
ALimit<-3
# gene has at least a variance of 0.5
varLimit<-0.5
# filter ExpressionSet
flist <- filterfun(function(x) var(x)>varLimit, kOverA(kLimit,ALimit))
exp.set.fil <- exp.set[genefilter(exp.set, flist), ]
```

Data normalization

Before clutsering the expression matrix is scaled and centered. Two normalized matrices are generated: the gene-wise (row-wise) normalized and the sample-wise (column-wise) normalized expression matrix.

```r
norm.ISA<-ISANormalize(exp.set.fil)
```

Iterative Bi-Clustering

The Algorithm starts with a random set of genes and will refine iteratively this input set. First those conditions are identified where the gene set exhibits a high correlation, then genes are removed/included that are well/poorly co-expressed within the selected conditions. This process is iterated until the gene expression signature is stable and does not change anymore in this process. This procedure is controled by two user-defined thresholds thr.feat and thr.sample, which define how closely related are the genes and the conditions respectively. We start with 300 randomly generated gene sets. Next ISA is applied and co-regulated modules are collected. Finally duplicated modules are removed.

```r
random.seed <- generate.seeds(length=nrow(norm.ISA), count=3)
#Bi-Clustering
module_run <- ISAIterate(norm.ISA, feature.seeds=random.seed,
                         thr.feat=1.75, thr.samp=0.45, convergence="cor")
# Remove duplicated modules
modules.unique <- ISAUnique(norm.ISA, module_run)
```

Session info

```r
sessionInfo()
```

```
## R version 3.3.1 (2016-06-21)
## Platform: x86_64-apple-darwin13.4.0 (64-bit)
## Running under: OS X 10.12.6 (Sierra)
##
## locale:
## [1] de_DE.UTF-8/de_DE.UTF-8/de_DE.UTF-8/C/de_DE.UTF-8/de_DE.UTF-8
##
## attached base packages:
## [1] stats4    parallel  stats     graphics  grDevices utils     datasets
## [8] methods   base
##
## other attached packages:
## [1] org.Hs.eg.db_3.3.0   genefilter_1.54.2    eisa_1.24.0
## [4] AnnotationDbi_1.34.4 IRanges_2.6.1        S4Vectors_0.10.3
## [7] Biobase_2.32.0       BiocGenerics_0.18.0  isa2_0.3.5
##
## loaded via a namespace (and not attached):
##  [1] Rcpp_0.12.12    bitops_1.0-6    tools_3.3.1     digest_0.6.12
##  [5] bit_1.1-12      annotate_1.50.1 RSQLite_2.0     evaluate_0.10.1
##  [9] memoise_1.1.0   tibble_1.3.3    lattice_0.20-35 pkgconfig_2.0.1
## [13] rlang_0.1.2     Matrix_1.2-11   graph_1.50.0    DBI_0.7
## [17] Category_2.38.0 yaml_2.1.14     stringr_1.2.0   knitr_1.17
## [21] rprojroot_1.2   bit64_0.9-7     grid_3.3.1      GSEABase_1.34.1
## [25] survival_2.41-3 XML_3.98-1.9    RBGL_1.48.1     rmarkdown_1.6
## [29] blob_1.1.0      magrittr_1.5    splines_3.3.1   backports_1.1.0
## [33] htmltools_0.3.6 xtable_1.8-2    stringi_1.1.5   RCurl_1.95-4.8
```

TCGA colon/adenocarcinoma data analysis

Uwe Schwartz

20 8 2017

Dependencies

```
library(multtest)
```

Load data

This markdown script contains code to compare weather identifed Evi/Wls non-canonical regulated genes are correlated with mRNA expression of Evi/Wls in colon cancer (TCGA date set, 2013). Level 3 microarray expression data was downloaded from TCGA data portal (https://tcga-data.nci.nih.gov/tcga/).

```
# function to load TCGA data into R
loadTCGA<-function(path){
  norm.mx<-list.files(path)
  for(i in 1:length(norm.mx)){
    part<-read.delim(paste0(path,norm.mx[i]))
    if(i==1){
      exp.ma<- as.matrix(as.numeric(as.character(part$value)))
      rownames(exp.ma)<-as.character(part$gene.symbol)
      colnames(exp.ma)<- as.character(part$barcode[1])
    } else {
    exp.ma<- cbind(exp.ma, as.numeric(as.character(part$value)))
    colnames(exp.ma)[i]<-  as.character(part$barcode[1])
    }
  }
  return(exp.ma)
}

# load matched normal tissue data (n=12)
path.1<-paste0("data/TCGA/8957cb58-4ae8-44e4-b52f-0405cb175a85/",
               "Expression-Genes/UNC__AgilentG4502A_07_3/Level_3/")
ma.1<-loadTCGA(path.1)

# load unmatched normal tissue data (n=7)
path.2 <-paste0("data/TCGA//a304baf7-96d7-445c-b9bf-b475bcf3fa4e/",
                "Expression-Genes/UNC__AgilentG4502A_07_3/Level_3/")
ma.2<-loadTCGA(path.2)

# load tumor tissue data (n=155)
path.3 <-paste0("data/TCGA/21c03af6-e84d-469d-9798-e2563e0cce5a/",
                "Expression-Genes/UNC__AgilentG4502A_07_3/Level_3/")
ma.3<-loadTCGA(path.3)

# merge data sets,
exp.ma<-cbind(ma.1,ma.2,ma.3)
```

Differential expression analysis

Next we selected samples with high differences in EVI expression, which is involved in Wnt secretion. In this analysis we assume that in Samples with low EVI expression, complete Wnt secretion and hence signaling is impaired compared to samples with high EVI expression.

```
# select EVI (=GPR177)
evi.exp<-exp.ma[rownames(exp.ma)=="GPR177",]

# select samples, whose EVI expression differs more than 1 standard deviation from median
up.reg<-evi.exp>(median(evi.exp)+1*sd(evi.exp))
down.reg<-evi.exp<(median(evi.exp)-1*sd(evi.exp))

evi.up<-evi.exp[up.reg]
evi.down<-evi.exp[down.reg]
```

We can now visualize the distribution of Evi expression and the selection thresholds for further analysis. Thresholds are indicated by red lines.

```
hist(evi.exp, breaks=30, xlim=c(-3,2), xlab="Rel. expression",
     main="EVI Expression in TCGA data set")
abline(v=median(evi.exp)+c(1,-1)*sd(evi.exp), col="red")
```

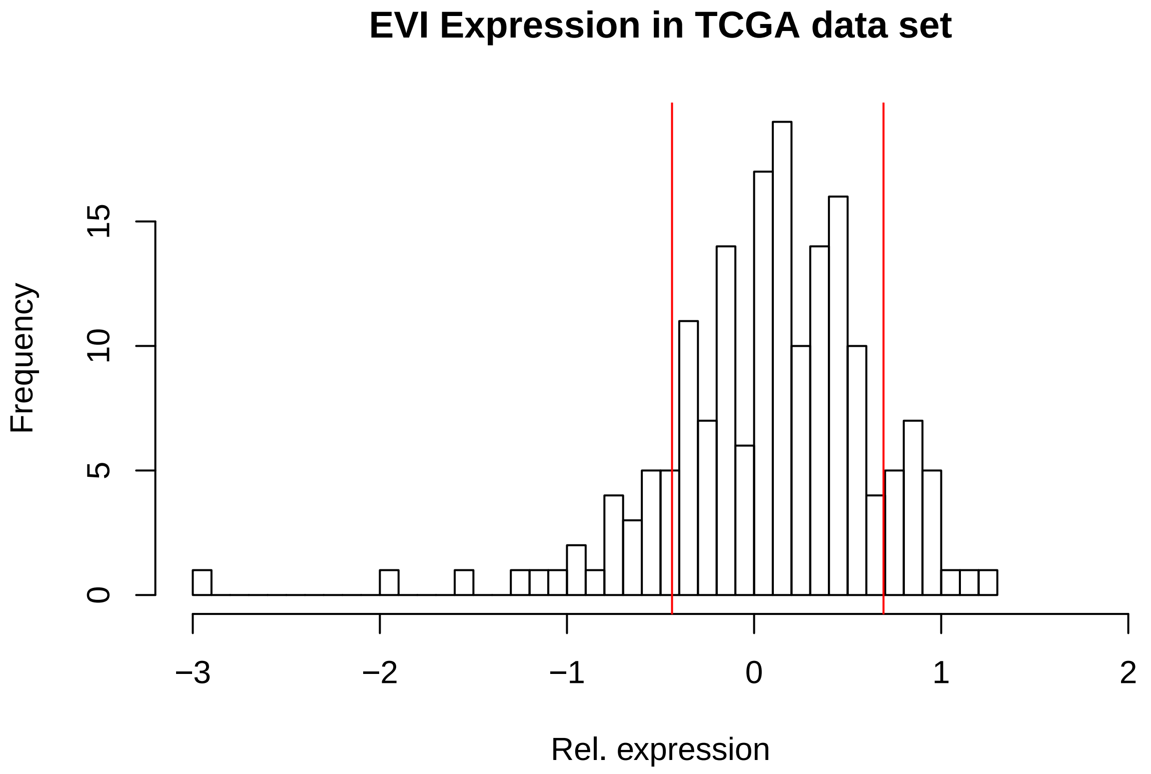

Statistical test

We use a t-test statistic to identify differentially expressed genes between low EVI samples and high EVI samples. Finally p-values are corrected for multiple testing.

```r
ttest.data<-exp.ma[,c(names(evi.up), names(evi.down))]
label<- c(rep(1,length(evi.up)), rep(0, length(evi.down)))
# T-test
tStat<- mt.teststat(ttest.data, classlabel=label, test="t")
# get p-value
ttest.p<-2*pt(-abs(tStat), df=(ncol(ttest.data)-2))
# plot p-value distribution
hist(ttest.p, breaks=20, xlab="p-value", main="p-value distribution")
```

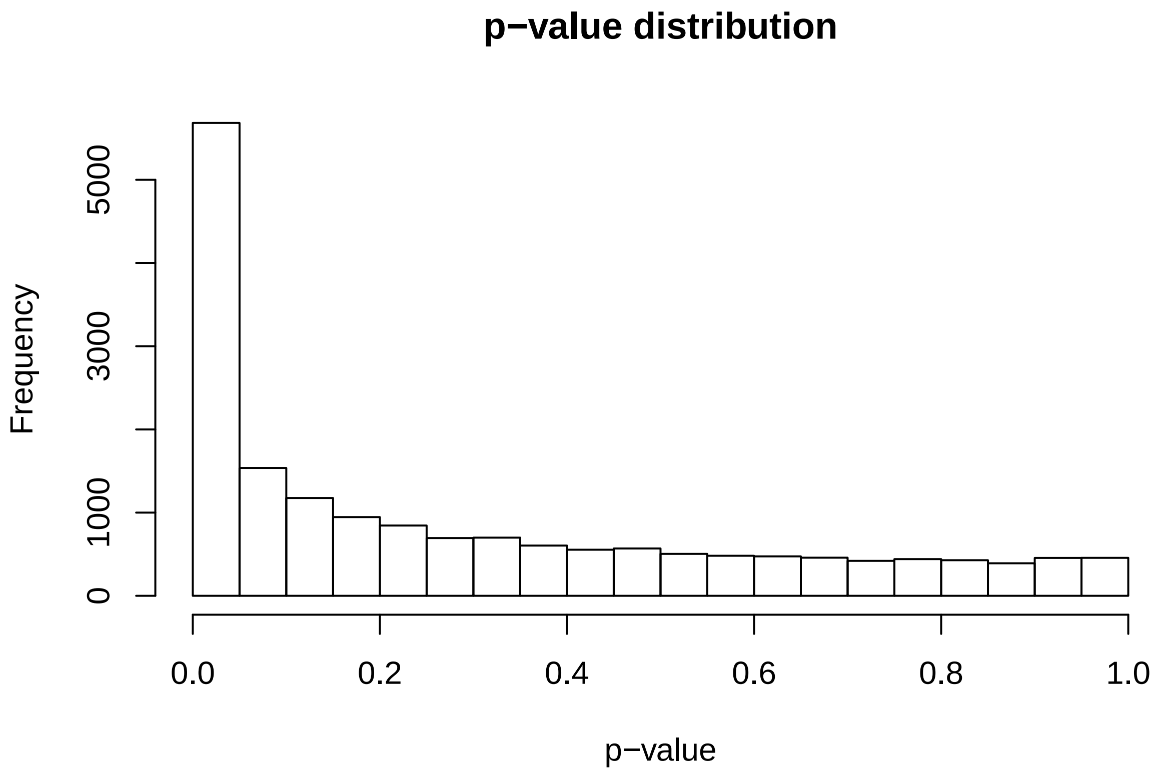


```r
# adjust p-Value for multiple testing by Benjamini-Hochberg
pAdjusted <- mt.rawp2adjp(ttest.p, proc = c("BH"))
```

In the last step a final table is generated comprised of within-group median expression levels, the between-group fold change and the raw/adjusted p-values.

```r
# assign gene names
pAdj<-(pAdjusted$adjp[order(pAdjusted$index),])
rownames(pAdj)<- rownames(ttest.data)

#calculate logFC
low.EVI.median<-apply(ttest.data[,label==0], 1, median)
high.EVI.median<-apply(ttest.data[,label==1], 1, median)
# calculate fold change
fc.high_low<- 2**(low.EVI.median-high.EVI.median)
# final table
table.EVI<-cbind(low.EVI.median, high.EVI.median, fc.high_low, pAdj)
colnames(table.EVI)[1:3]<- c("low EVI - TCGA", "high EVI - TCGA",
                             "FoldChange [lowEVI/highEVI]")
```

Session info

```r
sessionInfo()
```

```
## R version 3.3.1 (2016-06-21)
## Platform: x86_64-apple-darwin13.4.0 (64-bit)
## Running under: OS X 10.12.6 (Sierra)
##
## locale:
## [1] de_DE.UTF-8/de_DE.UTF-8/de_DE.UTF-8/C/de_DE.UTF-8/de_DE.UTF-8
##
## attached base packages:
## [1] parallel  stats     graphics  grDevices utils     datasets  methods
## [8] base
##
## other attached packages:
## [1] multtest_2.28.0     Biobase_2.32.0      BiocGenerics_0.18.0
##
## loaded via a namespace (and not attached):
##  [1] Rcpp_0.12.12    lattice_0.20-35 digest_0.6.12   rprojroot_1.2
##  [5] MASS_7.3-47     grid_3.3.1      backports_1.1.0 stats4_3.3.1
##  [9] magrittr_1.5    evaluate_0.10.1 stringi_1.1.5   Matrix_1.2-11
## [13] rmarkdown_1.6   splines_3.3.1   tools_3.3.1     stringr_1.2.0
## [17] yaml_2.1.14     survival_2.41-3 htmltools_0.3.6 knitr_1.17
```
